# Supplementary figures and images for: Translating staff experience into organisational improvement: the HEADS-UP stepped wedge, cluster controlled, non-randomised trial
Source: BMJ Open. 2017 Jul 18;7(7):e014333. doi: 10.1136/bmjopen-2016-014333 (PMC5541585; doi:10.1136/bmjopen-2016-014333)

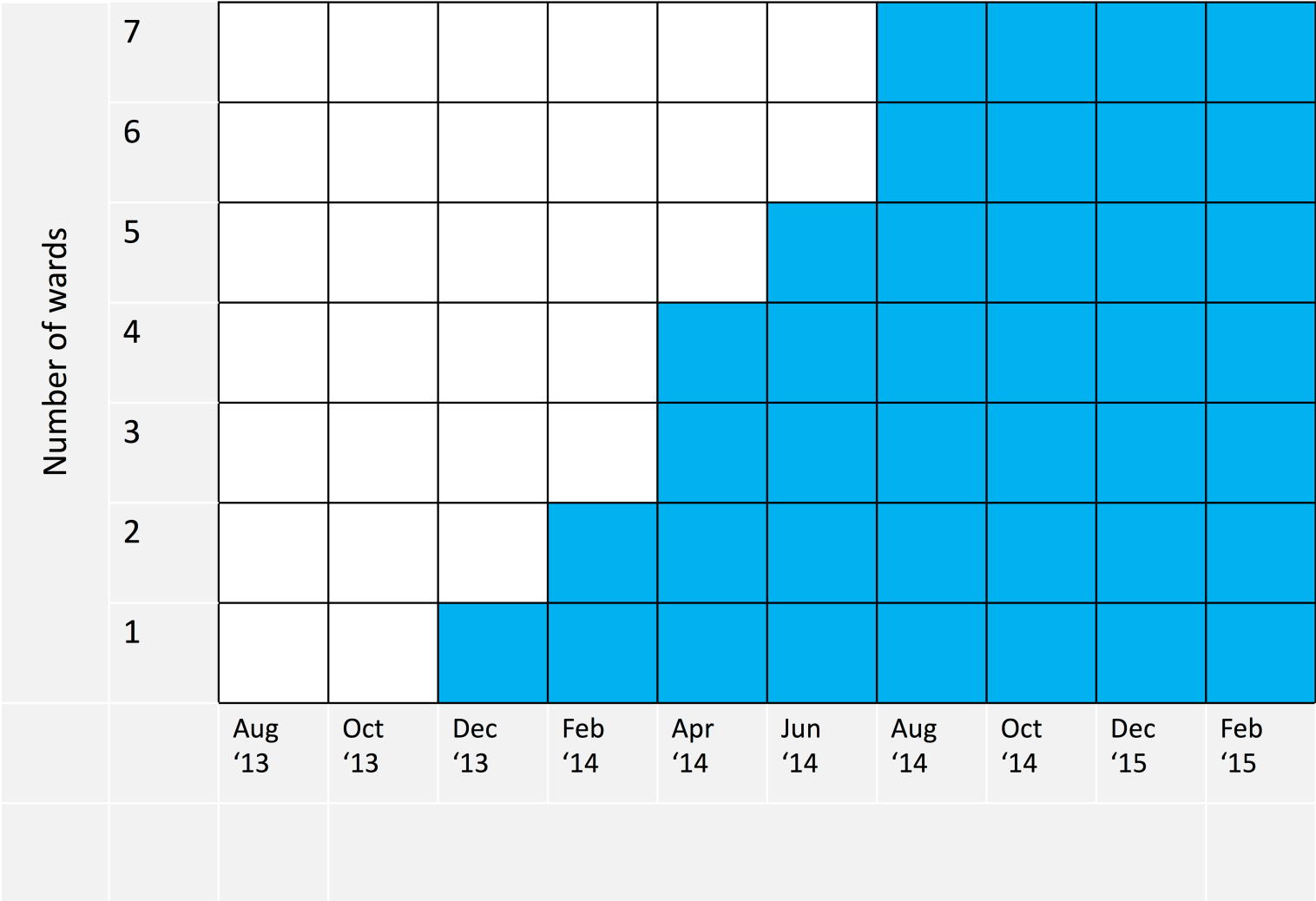

Supplement: Supplementary Figure 1 [file bmjopen-2016-014333supp001.pdf]
