# Supplementary material for: Translating staff experience into organisational improvement: the HEADS-UP stepped wedge, cluster controlled, non-randomised trial
Source: BMJ Open. 2017 Jul 18;7(7):e014333. doi: 10.1136/bmjopen-2016-014333 (PMC5541585; doi:10.1136/bmjopen-2016-014333)
Supplement: Supplementary Figure 2 [file bmjopen-2016-014333supp002.pdf]

What happened yesterday?

HEADS-UP

A first class hospital for our community

Design by HELIX
helixcentre.com

If a patient has been affected, please include their hospital number. Where there is nothing to report, write N/A in comments box.

1 Review details

Ward:

Scribe name: Role:

Date:

Team members present

- Clerk
- HCA
- Physio
- OT
- Nurse
- Pharmacist
- Discharge team
- Consultant
- SpR
- CT
- F2
- F1

2 Concerns

Staffing levels

- Medical
- Nursing
- OT / PT

Comment or N/A

Equipment

- Unavailable
- Not working

Comment or N/A

Services

- Phlebotomy
- Echo
- Radiology
- Endoscopy
- In-patient review
  - Delayed
  - Too junior

Comment or N/A

Missed results / diagnosis

Comment or N/A

3 Events

Handover / communication problem

Comment or N/A

New hospital-acquired problem

- Pressure ulcer
- Pneumonia
- UTI
- MRSA
- C-Diff
- Other

Comment or N/A

Patient deterioration

- Sepsis
- Delirium
- Fall
- Arrhythmia
- Seen by medical team
- Seen by outreach / ICU

Comment or N/A

Death

- Expected
- Unexpected

Comment or N/A

Patient not discharged as expected

Comment or N/A

4 Drug errors and other issues

- Prescription
- Administration
- VTE
- Analgesia
- Antibiotics
- Nutrition

Comment or N/A

5 What went really well?

Comment or N/A

6 Who will...

Arrange senior review / outreach for sick patient?	Name or N/A
Change a prescription?	Name or N/A
Escalate equipment problem / discharge delay?	Name or N/A
Complete a DATIX?	Name or N/A
Log patient for M&M discussion?	Name or N/A
Other?	Name or N/A
