# Supplementary material for: Translating staff experience into organisational improvement: the HEADS-UP stepped wedge, cluster controlled, non-randomised trial
Source: BMJ Open. 2017 Jul 18;7(7):e014333. doi: 10.1136/bmjopen-2016-014333 (PMC5541585; doi:10.1136/bmjopen-2016-014333)
Supplement: Supplementary Table 1 [file bmjopen-2016-014333supp003.pdf]

Supplement Table 1: survey respondents and completion rates

Variable	Baseline	6 months
Professional background [n (%)]		
Consultant physician	8 (13.1)	5 (9.3)
Junior physician	28 (45.9)	35 (64.8)
Senior nurse	15 (24.6)	7 (13.0)
No answer	10 (16.4)	7 (13.0)
Median time on the ward [months]		
	4-6	0-3
Ward specialty [n (%)]		
Acute medicine	19 (31.1)	10 (18.5)
Gastroenterology & general medicine	13 (21.3)	11 (20.4)
Heart failure & general medicine	4 (6.6)	5 (9.3)
Geriatrics	16 (26.2)	14 (25.9)
Respiratory & general medicine	9 (14.8)	12 (22.2)
No answer	0	2 (3.7)
Participated in more than 5 HEADS-UP briefings [n (%)]		
Yes	13 (21.3)	37 (68.5)
No	47 (77.0)	14 (25.9)
No answer	1 (1.6)	3 (5.6)
Workload perception score (NASA-TLX) [mean (SD)]		
	13.2 (2.7)	12.8 (3.1)
Unscored safety and teamwork items [n (%)]		
	33/1647 (2.0)	37/1458 (2.5)
Unscored NASA-TLX items [n (%)]		
	21/366 (5.7)	(18/324) (5.6)
