# Supplementary material for: Translating staff experience into organisational improvement: the HEADS-UP stepped wedge, cluster controlled, non-randomised trial
Source: BMJ Open. 2017 Jul 18;7(7):e014333. doi: 10.1136/bmjopen-2016-014333 (PMC5541585; doi:10.1136/bmjopen-2016-014333)
Supplement: Author's manuscript [file bmjopen-2016-014333.draft_revisions.pdf]

BMJ Open

Translating staff experience into organisational improvement: the HEADS-UP stepped wedge cluster controlled trial

Journal:	*BMJ Open*
Manuscript ID	bmjopen-2016-014333
Article Type:	Research
Date Submitted by the Author:	17-Sep-2016

Complete List of Authors:	Pannick, Samuel; Imperial College London, NIHR Imperial Patient Safety Translational Research Centre<br>Athanasiou, Thanos; Imperial College London, Surgery and Cancer<br>Long, Susannah; Imperial College London, Centre for Patient Safety and Service Quality<br>Beveridge, Iain; West Middlesex University Hospital NHS Trust, Department of Medicine<br>Sevdalis, Nick; King's College London,

**Primary Subject Heading**:	Health services research
Secondary Subject Heading:	Medical management
Keywords:	patient safety, Quality in health care < HEALTH SERVICES ADMINISTRATION & MANAGEMENT, stepped wedge, medical ward, prospective clinical surveillance

SCHOLARONE™ Manuscripts

For peer review only

'Translating staff experience into organisational improvement: the HEADS-UP stepped wedge cluster controlled trial'

Samuel Pannick, MA MBBS, MRCP[1]; Thanos Athanasiou, MD, PhD[2]; Susannah J Long, MA MB BS, FRCP, PhD[1,3]; Iain Beveridge, MB, BChir, FRCP[4]; and Nick Sevdalis, PhD[5]

[1] NIHR Imperial Patient Safety Translational Research Center, Imperial College London, London, UK

[2] Department of Surgery & Cancer, Imperial College London, London, UK

[3] Imperial College Healthcare NHS Trust, London, UK

[4] West Middlesex University Hospital NHS Trust, Isleworth, UK

[5] Center for Implementation Science, King's College London, London, UK

Trial registration: ISRCTN 34806867 (http://www.isrctn.com/ISRCTN34806867)

Corresponding author:

Dr Sam Pannick, MA MB BS MRCP

Room 503 Medical School Building, Norfolk Place, London W2 1PG. United Kingdom

s.pannick@imperial.ac.uk; + (44) 207 3543149

Key words: interdisciplinary care; medical wards; patient safety; stepped wedge trial

Contributions:

Study design: SP, TA, NS. Study implementation: SP, SJL, IB. Statistical analysis: SP, TA. All authors contributed to, read and approved the final manuscript. Dr Pannick had full access to all of the data in the study and takes responsibility for the integrity of the data and the accuracy of the data analysis.

Financial support:

This paper represents independent research supported by the National Institute for Health Research (NIHR) Imperial Patient Safety Translational Research Centre, Imperial College Healthcare Charity (Grant GG14\1022), and West Middlesex

For peer review only

University Hospital NHS Trust. NS' research is supported by the NIHR Collaboration for Leadership in Applied Health Research and Care South London at King's College Hospital NHS Foundation Trust. NS is a member of King's Improvement Science, which is part of the NIHR CLAHRC South London and comprises a specialist team of improvement scientists and senior researchers based at King's College London. Its work is funded by King's Health Partners (Guy's and St Thomas' NHS Foundation Trust, King's College Hospital NHS Foundation Trust, King's College London and South London and Maudsley NHS Foundation Trust), Guy's and St Thomas' Charity, the Maudsley Charity and the Health Foundation. NS is the director of London Training & Safety Solutions Ltd, which delivers team assessment and training to hospitals on a consultancy basis. No funding source had any role in the design and conduct of the study; collection, management, analysis or interpretation of the data; or preparation, review or approval of the manuscript. The views expressed are those of the authors and not necessarily those of the NHS, the NIHR or the Department of Health.

Abstract length: 299 words

Manuscript length: 3,135 words

Tables: 4

Figures: 1

Online content: 2 figures; 1 table

For peer review only

ABSTRACT

Objectives:

Frontline staff insights into care delivery correlate with patients' clinical outcomes. These outcomes might be improved through the near-real time identification and mitigation of staff concerns. We aimed to determine the effects of a prospective frontline surveillance system on patient and interdisciplinary team outcomes.

Design:

Prospective, stepped wedge, cluster controlled trial with mixed methods evaluation, conducted between December 2013 and February 2015.

Participants

Seven interdisciplinary medical ward teams, from two hospitals in the United Kingdom.

Intervention

Prospective clinical team surveillance (PCTS) – structured daily interdisciplinary briefings to capture staff-perceived safety threats, with organisational facilitation and feedback.

Main measures

The primary outcome was excess length of stay (eLOS): an admission more than 24 hours longer than the local average for comparable patients. Secondary outcomes included in-hospital death or death/readmission within 30 days, and team outcomes (safety and teamwork climates, and incident reporting). Safety and teamwork climates were measured with the Safety Attitudes Questionnaire.

For peer review only

Results

Briefings were performed on 74.2% working days/month (range 15–100%), documenting 80.7 issues/ward/month (range 6–208). 11,445 patients were included in the primary analysis (control group, n=4,927; intervention group, n=6,518). In an unadjusted (intention-to-treat) model, PCTS had no impact on eLOS (OR=1.07, 95% CI 0.96-1.19, p=0.219). In an adjusted (per protocol) analysis, high fidelity PCTS reduced eLOS (OR=0.78, 95% CI 0.66–0.94, p=0.007). Other patient outcomes were unchanged. High fidelity implementation increased the total number of incident reports submitted each month, with increases in non-falls incident reports (IRR 1.41, 95% CI 1.18-1.69, p<0.001) and reports from non-nursing staff (IRR 1.60, 95% CI 1.08-2.36, p=0.019). Safety and teamwork climates improved with sustained PCTS.

Conclusions

High fidelity PCTS implementation reduced eLOS for general medical patients, and improved interdisciplinary team outcomes. Future studies should focus on improving the implementation of prospective frontline surveillance strategies, to realise their full benefits.

For peer review only

STRENGTHS AND LIMITATIONS OF THIS STUDY

This is the first formal evaluation of a prospective frontline surveillance strategy on general medical wards.

The study was well powered to detect a significant change in its primary outcome, complemented by a mixed methods assessment of secondary patient and clinical team outcomes.

The pragmatic nature of the trial increases the generalisability of its findings.

It can be difficult to disentangle the effects of interdisciplinary interventions from the characteristics of the teams that implement them best, although our statistical model allowed for varying intervention fidelity by each team over the course of the study.

Contamination between the groups, whereby additional organisational support was directed to those wards in the control period, may have reduced the intervention's measurable effect.

INTRODUCTION

Frontline staff have a particular insight into the safety and quality of inpatient care. Favorable staff perceptions of care correlate with improved clinical outcomes, including the likelihood of patient survival and harm-free care.[1,2] Yet frontline concerns about the practical delivery of care do not feature in national safety initiatives, nor are they a priority at a local level.[3,4] General medical wards, in particular, struggle to mobilise organisational resources – even though preventable hospital deaths are disproportionately caused by ward failings.[5]

Hospital leaders acknowledge the need to access frontline insights, especially those that might challenge their assumptions about organisational performance.[6] Yet leaders are wary of acting too quickly, for fear of wasting resources on unvalidated concerns.[6] At the same time, actionable quality and safety concerns – identified by

For peer review only

medical teams in the course of their normal work[7-9] – remain hidden from organisational view. These concerns are typically addressed by the teams themselves, using superficial workarounds that allow clinical work to continue but do not prevent the problem's recurrence.[10] Temporary fixes are encouraged by a professional pride in troubleshooting,[11] and organisational traditions that emphasise individual vigilance.[10] Better methods are needed to capitalise on frontline experiences and translate them into organisational action.

An ideal system for effective resolution of frontline concerns would therefore: (i) promote the systematic identification of safety concerns by frontline staff; (ii) motivate and enable them to resolve unit-level issues within their control; (iii) record frontline successes and challenges to build a richer understanding of safety and resilience across the organisation; and (iv) engage leadership in attending to frontline concerns. We aimed to achieve these goals through prospective clinical team surveillance (PCTS) – a program combining structured, frontline interdisciplinary briefings with facilitated organisational escalation of the issues they identified, and feedback. The effects of interdisciplinary team interventions in general medical settings have been mixed,[12,13] and recent editorials have called for adequately powered studies with designs that better account for confounding factors.[14] Here, we evaluate PCTS in a pragmatic, stepped wedge, cluster controlled trial, reporting its effects on patient outcomes, escalation of care, and staff safety attitudes and behaviors.

METHODS

Intervention program

i) Structured team briefing

The PCTS program was based on published observational studies showing that medical teams can engage in the rapid identification and review of potential adverse events.[7,15-18] We designed a daily interdisciplinary briefing for structured team self-report on medical wards: the HEADS-UP (Hospital Event Analysis Describing Significant Unanticipated Problems) briefing. HEADS-UP briefings identified clinical and administrative challenges of the preceding 24 hours. They centered on the

For peer review only

problems identified most commonly in the medical ward setting, as described in a recent, large, observational European study.[19] Structured team self-report captures the majority of errors documented by a trained observer.[20]

HEADS-UP briefings were co-developed with frontline clinical staff between October and November 2013. At the request of clinicians, a pro forma was developed with a visual format similar to the World Health Organisation's surgical safety checklist[21] [online Figure 1]. Best practice recommendations for improvement interventions advise a degree of local flexibility:[22] other teams were able to tailor their unit's pro forma whilst retaining the overall format. Briefings ended with a team agreement on how to resolve, or escalate, the concerns that had been discussed. After a short period of supervision by the program lead (a resident [S.P.] with a 0.75 full time equivalent commitment, working as an `embedded researcher'[23]), the briefing was devolved to the ward team. Teams introduced daily HEADS-UP briefings between Monday and Friday, at the most appropriate time in their usual workflow. There was no protected time for team training. Briefings could be led by any member of the ward team, from ward clerk to therapist to attending physician.

ii) Facilitation

The second component of the intervention was facilitation, advancing the issues raised in the HEADS-UP briefings to bring about tangible unit-level and organisational changes. Facilitation helps to make sense of quality improvement interventions, aligning them with their participants and the surrounding context.[24] It is necessarily opportunistic and malleable, taking advantage of existing organisational levers. Here, it involved (for example) working with frontline teams to identify and document their areas of concern more effectively; championing those concerns in meetings with service leaders and safety committees; and following up on subsequent agreed actions when other priorities threatened their resolution. Other studies that have used a facilitator in a similar way have described the role as an '*animateur*', bringing on board people over whom he has no direct managerial authority.[25,26]

For peer review only - http://bmjopen.bmj.com/site/about/guidelines.xhtml

iii) Feedback

The third component of the intervention was feedback to participating teams, managers, governance committees, and senior executives. Feedback summarised and disseminated the information collected in the daily briefings, highlighting HEADS-UP performance in each area, common concerns and challenges, and recurrent or unresolved problems that might require additional support. HEADS-UP data were provided on request to service leads, to support their business planning. System changes arising from HEADS-UP were publicised, e.g., in existing departmental meetings, and via email and posters. As much as possible, feedback delivery was timely, focused on solution-finding, signposted to relevant resources, and adopted a non-judgmental approach.[27] Facilitation and feedback were provided by the program lead.

Although described separately here, we hypothesised that the intervention components would be interlinked, in that changes arising from the program's facilitation and feedback would motivate increasing engagement with HEADS-UP briefings, in turn increasing their ability to bring about change. We anticipated that improvements in interdisciplinary team care effectiveness would be brought about by parallel improvements in ward teams' function, and incremental support service improvement in response to escalation of their concerns.

Study design and patients

As described in the published protocol,[28] this stepped wedge trial was conducted on seven medical wards at two UK hospitals (Table 1). Interdisciplinary ward teams were assigned to implement the intervention at intervals, such that by the end of the trial all teams had adopted the intervention and had contributed both control- and intervention-group data. Stepped wedge designs are increasingly used to evaluate service-level interventions, where beneficial effects are anticipated.[29] Baseline data collection began in August 2013, with introduction of the intervention between December 2013 and February 2015. The order in which wards adopted the intervention was pragmatically guided by logistical constraints, as described in previous stepped wedge evaluations.[30]

For peer review only - http://bmjopen.bmj.com/site/about/guidelines.xhtml

Adult patients admitted to participating wards during the study period were eligible. Exclusion criteria were: (1) less than 50% of the hospital admission spent on the specified ward; (2) discharge to a new skilled care facility (not the patient's previous address) or other hospital; (3) multiple ward transfers; (4) admission to the high dependency unit or ICU; (5) elective admission, or direct admission from another hospital; and (6) surgeon-directed care for more than 24 hours of the admission.

The study was approved as a quality improvement program by research and development authorities at participating institutions.

Study outcomes

The primary outcome was excess length of stay (eLOS). eLOS was a binary variable: an admission more than 24 hours above the local average for similar patients, as classified by their Healthcare Resource Groups.[31] Local institutions contributing comparator data were four nearby hospitals, broadly subject to the same community service restrictions and healthcare economy demands.

Secondary outcomes included patient-level measures of (i) readmission within 30 days and (ii) in-hospital death or death/readmission within 30 days. Ward-aggregate measures included (iii) escalation events (referrals to the ICU outreach service, emergency calls and ICU transfers); (iv) complications of care (pressure ulcers, C.difficile infections, and MRSA, MSSA and E.coli bacteremias); and (v) incident reporting characteristics. (vi) Safety and teamwork climates were assessed at baseline and six months into the intervention period, using the relevant subscales from the well-established Safety Attitudes Questionnaire.[32,33]

Sample size

Power calculations were conducted for the primary outcome (eLOS), using the recommended methodology for stepped wedge trials.[34] We adopted a conservative intraclass correlation coefficient of 0.06, two-sided p<0.05, and iterated calculations based on the wards with the highest and lowest baseline outcome rates.[28] With

For peer review only - http://bmjopen.bmj.com/site/about/guidelines.xhtml

seven wards admitting 7840 eligible patients (560 per month), the trial's power to detect a 2-14% absolute risk reduction lay between 75% and 100%.[28] Other stepped wedge trials have similarly produced a range within which the study power might lie.[35]

Allocation and blinding

Individual patients were not recruited separately to the study, so we did not anticipate significant bias due to lack of allocation concealment.[36] Staff could not be blinded to their ward's assignment, due to the nature of the intervention. Clinical outcome data and escalation of care data were extracted by local administrative staff blinded to the study, as part of their ordinary duties. Hospital peer group data were generated by *CHKS* (Alcester, UK) and *Dr Foster* (London, UK), also blinded to intervention group.

Statistical analysis

Multi-level mixed-effects models (Stata, v14.1) were used to evaluate the intervention's effect on each patient- and ward-level outcome. This statistical approach accounts for clustering of outcomes within wards, and repeated measurements over time, representing ward-level variance in patient outcomes as a random effect. For the binary patient-level outcomes, binary logistic models were used; for counts data (complications of care and processes of care), we used Poisson loglinear models. Analyses involving eLOS were restricted to those patients who survived to discharge; no patients were excluded on the basis of 'outlier' length of stay. General linear models were used to analyse survey data (SPSS, v22).

In addition to the intention-to-treat analysis, we conducted a per protocol analysis, evaluating the effect of briefing implementation fidelity. Based on observations during the pilot period, we described implementation fidelity each month as the product of briefing frequency and engagement. High fidelity required both high briefing frequency and correspondingly intensive documentation by the ward. Frequency was coded into three categories: high (≥75% working days that month), medium (50-75%), and low (<50%). Monthly engagement was defined as high

For peer review only

(documentation of more than the median number of issues) or low (below the median). An interaction term 'frequency*engagement' was incorporated into each model, with codes for high, moderate, low and poor monthly implementation fidelity. Note that this per protocol analysis, which calculated different implementation fidelity codes for each ward-month, allowed for varying fidelity by a single ward team over the course of the study.

Patient-level outcomes were adjusted for age, Charlson comorbidity index,[37] and palliation status, as well as ward admissions and temporal trends. Ward-aggregate outcomes were adjusted for seasonal and temporal trends, the median Charlson score of patients on the ward that month, and the rotation of interns between departments. Survey analysis defined HEADS-UP participation as self-reported engagement in 5 or more briefings, and adjusted for self-reported workload on the NASA Task Load Index scale,[38] as well as hospital site and time period.

RESULTS

12,077 eligible admissions took place during the study period, of which 11,445 were included in the primary analysis [patient flow diagram]. 4,927 admissions were in the control group and 6,518 admissions were in the intervention group. Monthly ward admissions were higher in the intervention months (77 admissions/ward/month *vs* 64 admissions/ward/month), as was unadjusted length of stay (2.25 days *vs* 2.0 days) (Table 2).

Implementation fidelity

Briefing implementation data were available for 71/73 (97.2%) ward months. On average, wards conducted briefings on 74.2% working days/month (range 15 – 100%), documenting 80.7 issues/ward/month (range 6 – 208). In the intervention group, 3607/6518 (55.3%) admissions were in high fidelity ward months, 990/6518 (15.2%) in moderate fidelity ward months, and 1921/6518 (29.5%) in poor fidelity ward months.

For peer review only

Successful implementation at hospital 1 was also recognised by the Care Quality Commission (the UK's healthcare quality inspectorate), whose inspectors observed briefings in use and formally reported them as 'outstanding practice', their highest rating. More limited organisational attention at site 2 resulted in slower program implementation. At that site, PCTS was only embedding into routine practice as the second survey period began.

Primary outcome: excess length of stay (eLOS)

1279/4927 (26.0%) control admissions had eLOS *vs* 1714/6518 (26.3%) intervention admissions (Table 3). In the unadjusted model, PCTS had no impact on eLOS (OR=1.07, 95% CI 0.96-1.19, p=0.219); in the adjusted intention-to-treat model, the intervention was associated with increasing eLOS. However, this did not reflect whether the intervention had actually been used.

Implementation fidelity significantly affected the likelihood of eLOS ($\chi^2 = 7.62$, p=0.0221). eLOS was significantly reduced with high fidelity PCTS (OR=0.78, 95% CI 0.66-0.94, p=0.007). This reflected an average (absolute) 2.3% reduction in eLOS, in the wards most often implementing PCTS with high fidelity. An exploratory plot of the relationship between engagement, briefing frequency, and the probability of eLOS reduction is shown [Figure 1].

Secondary outcomes

Changes in processes of care and incident reporting were also dependent on implementation fidelity. With moderate fidelity implementation, the composite number of escalation events each month increased (IRR 1.35, 95% CI 1.04-1.75, p=0.024). Further, high fidelity implementation increased the total number of incident reports submitted each month (IRR 1.33, 95% CI 1.15-1.55, p<0.001), with increases in non-falls incident reports (IRR 1.41, 95% CI 1.18-1.69, p<0.001) and reports from non-nursing staff (IRR 1.60, 95% CI 1.08-2.36, p=0.019). Complications of care, and other secondary outcomes, were unchanged.

For peer review only - http://bmjopen.bmj.com/site/about/guidelines.xhtml

Survey response rates were 71.8% (61/85) at baseline and 65.1% (54/83) at 6 months, with 97.7% safety and teamwork questions completed [online Table 1]. Unadjusted mean safety and teamwork scores were non-significantly higher after PCTS participation [Table 4]. With sustained implementation, PCTS improved adjusted safety and teamwork scores over time (site 1 estimated marginal mean [EMM] safety score 70.3 *vs* 61.0, p=0.021; EMM teamwork score 81.7 *vs* 70.0, p=0.004). 71.9% (23/32) of site 1 respondents agreed they had reported concerns differently as a result of the programme. Of note, higher workload scores correlated with lower safety scores (B= -1.52, 95% CI -2.59 – -0.44, p=0.006) but not teamwork scores (B= -0.21, 95% CI -1.10 – 0.68, p= 0.64).

DISCUSSION

In this stepped wedge cluster controlled trial, high fidelity PCTS implementation reduced eLOS. Significant improvement was seen in per protocol, but not intention-to-treat, analyses. Amongst the secondary outcomes, safety and teamwork climates improved with sustained implementation, with some evidence of change in the processes of escalation of care. PCTS increased incident reporting by non-nursing staff, and the number of non-falls reports. Both suggest improved frontline engagement with safety reporting. Ordinarily, few incident reports are submitted by physicians,[39] and a higher proportion of non-falls incidents is associated with improved safety culture and risk management ratings.[40]

Conflicting intention-to-treat and per protocol evaluations for the primary outcome were unexpected. Though some quality improvement efforts may result in worse outcomes, perhaps by distracting attention from existing good practice,[41,42] our assessment of implementation consistently showed that better use of the intervention did lead to clinical improvement. There are two possible explanations for the discrepancy. First, there may have been unmeasured confounders later in the study period, when the intervention group (by design) accrued more data. Inclusion of fixed effects for time and ward admissions may not have adequately adjusted for worsening health economy pressures and an increasingly high threshold for hospital admission. Ward admissions are an imperfect proxy for increasing workload:

For peer review only

demands on staff increase as the numbers of admissions, transfers and discharges all increase.[43] The effect of the intervention (when applied at low fidelity) would therefore be confounded by these changes.

Second, it is possible that underuse of the HEADS-UP briefings in some areas was a marker of wider team dysfunction and poor performance, rather than itself directly leading to poorer outcomes. Teams that participate more wholeheartedly in trials may be more innovative, with strong leadership, a readiness for change, and better managerial relations.[44] It is difficult to entirely separate the intervention's effects from the characteristics of the teams that implemented it best. Nonetheless, our statistical model accounted for ward-level variation in outcomes, and allowed the same team to contribute data to different categories of implementation fidelity, depending on their actions each month. Direct observation also showed multiple teams using briefings meaningfully, even during periods of great pressure. It is unlikely, therefore, that our results are a simple reflection of teams' pre-existing practice or fluctuations in their workload. However, interdisciplinary norms almost certainly played a role in how the intervention was perceived and adopted, and ultimately whether or not it was beneficial.

Few controlled studies have focused specifically on structures for interdisciplinary medical ward care.[12,45] In a systematic review, 70% of interdisciplinary interventions had no effect on the length of stay;[12] those that did were typically high intensity, facilitated interventions like PCTS. The mechanisms by which these interventions might improve outcomes remain poorly understood. Interdisciplinary team structures generate team processes and team outcomes, which produce patient outcomes;[46-48] however, better patient outcomes also reciprocally influence safety attitudes and behaviors, and attract managerial attention.[14,49] Nonetheless, we identified possible intermediate routes through which PCTS may have taken effect, with changes in teamwork and safety climates, and organisational efforts to remove barriers to frontline workflow. We have theorised that PCTS was most effective when the incentives for managers and clinical staff aligned.[50]

For peer review only - http://bmjopen.bmj.com/site/about/guidelines.xhtml

PCTS implementation in this real-world setting compared favorably to other structured team initiatives. Even when mandated, only 62% of surgical checklists are completed in their entirety, and as we found here, improved outcomes are detectable only when the intervention is completed in full.[51] Similarly, structured bedside rounds may be implemented for only 54% of patients, despite co-creation with frontline staff.[52] Wider adoption of PCTS may therefore be feasible, with appropriate attention to managerial engagement and implementation efforts.[50] In an overview of future patient safety efforts, Vincent and Amalberti called for more strategies that lend structure to teams' *ad hoc* decision-making, particularly in fluid and unpredictable settings like acute medical wards.[53] Our results suggest these strategies may contribute both to safety and efficiency, which need not be opposing values.[3,54]

This study's strengths include its pragmatism: the trial had broad eligibility criteria; a typical clinical setting; little additional investment for study recruitment or follow-up; and flexibility with regard to intervention delivery and adherence.[55] Limitations include the difficult balancing act between trial rigor and implementation effectiveness. Preserving distinct intervention and control groups may have constrained implementation fidelity, limiting a more natural, collaborative spread of practice. Contamination between the groups may have reduced the intervention's measurable effect: PCTS-generated organisational support (over which we had no control) was not necessarily directed to areas that first raised concerns. Complex interventions may be difficult to replicate in other settings, and these programs are particularly susceptible to funding cuts, even where they have been beneficial.[56] Forthcoming qualitative and implementation evaluations will identify how the intervention might best be adapted in the future.

CONCLUSIONS

High fidelity PCTS implementation reduced eLOS for general medical patients, without affecting other clinical outcomes. Other benefits included improved team safety and teamwork attitudes, and increases in high-yield elements of incident reporting. PCTS, when implemented well, may be an effective interdisciplinary team

For peer review only - http://bmjopen.bmj.com/site/about/guidelines.xhtml

structure on medical wards. Future work should focus on improving the quality of its implementation, to realise its full benefits.

ACKNOWLEDGEMENTS

This paper represents independent research supported by the National Institute for Health Research (NIHR) Imperial Patient Safety Translational Research Centre, Imperial College Healthcare Charity (Grant GG14\1022), and West Middlesex University Hospital NHS Trust. NS' research is supported by the NIHR Collaboration for Leadership in Applied Health Research and Care South London at King's College Hospital NHS Foundation Trust. NS is a member of King's Improvement Science, which is part of the NIHR CLAHRC South London and comprises a specialist team of improvement scientists and senior researchers based at King's College London. Its work is funded by King's Health Partners (Guy's and St Thomas' NHS Foundation Trust, King's College Hospital NHS Foundation Trust, King's College London and South London and Maudsley NHS Foundation Trust), Guy's and St Thomas' Charity, the Maudsley Charity and the Health Foundation. No funding source had any role in the design and conduct of the study; collection, management, analysis or interpretation of the data; or preparation, review or approval of the manuscript. The views expressed are those of the authors and not necessarily those of the NHS, the NIHR or the Department of Health. An earlier version of this work was presented at the Society of Hospital Medicine Annual Meeting 2016.

CONFLICTS OF INTEREST

NS is the director of London Training & Safety Solutions Ltd, which delivers team assessment and training to hospitals on a consultancy basis.

REFERENCES

1. Lawton R, O'Hara JK, Sheard L, et al. Can staff and patient perspectives on hospital safety predict harm-free care? An analysis of staff and patient survey data and routinely collected outcomes. BMJ Qual Saf 2015;**24**(6):369-76.

For peer review only

2. Tvedt C, Sjetne IS, Helgeland J, et al. An observational study: associations between nurse-reported hospital characteristics and estimated 30-day survival probabilities. BMJ Qual Saf 2014;**23**(9):757-64.

3. Tucker AL, Singer SJ, Hayes JE, et al. Front-line staff perspectives on opportunities for improving the safety and efficiency of hospital work systems. Health Serv Res 2008;**43**(5 Pt 2):1807-29.

4. Singer SJ, Gaba DM, Falwell A, et al. Patient safety climate in 92 US hospitals: differences by work area and discipline. Med Care 2009;**47**(1):23-31.

5. Hogan H, Healey F, Neale G, et al. Preventable deaths due to problems in care in English acute hospitals: a retrospective case record review study. BMJ Qual Saf 2012;**21**(9):737-45.

6. Martin GP, McKee L, Dixon-Woods M. Beyond metrics? Utilizing 'soft intelligence' for healthcare quality and safety. Soc Sci Med 2015;**142**:19-26.

7. Andrews LB, Stocking C, Krizek T, et al. An alternative strategy for studying adverse events in medical care. Lancet 1997;**349**(9048):309-13.

8. Lamba AR, Linn K, Fletcher KE. Identifying patient safety problems during team rounds: an ethnographic study. BMJ Qual Saf 2014;**23**(8):667-9.

9. Vincent C, Burnett S, Carthey J. The measurement and monitoring of safety. London: Health Foundation, 2013.

10. Tucker AL, Edmondson AC. Why hospitals don't learn from failures: Organizational and psychological dynamics that inhibit system change. California Management Review 2003;**45**(2):55-72.

11. Jeffs L, Berta W, Lingard L, et al. Learning from near misses: from quick fixes to closing off the Swiss-cheese holes. BMJ Qual Saf 2012;**21**(4):287-94.

For peer review only

12. Pannick S, Davis R, Ashrafian H, et al. Effects of Interdisciplinary Team Care Interventions on General Medical Wards: A Systematic Review. JAMA Intern Med 2015;**175**(8):1288-98.

13. Bhamidipati VS, Elliott DJ, Justice EM, et al. Structure and outcomes of interdisciplinary rounds in hospitalized medicine patients: A systematic review and suggested taxonomy. Journal of Hospital Medicine 2016;10.1002/jhm.2575:n/a-n/a.

14. O'Leary KJ, Johnson JK, Auerbach AD. Do interdisciplinary rounds improve patient outcomes? Only if they improve teamwork. Journal of Hospital Medicine 2016;**11**(7):524-5.

15. Forster AJ, Fung I, Caughey S, et al. Adverse events detected by clinical surveillance on an obstetric service. Obstet Gynecol 2006;**108**(5):1073-83.

16. Forster AJ, Kyeremanteng K, Hooper J, et al. The impact of adverse events in the intensive care unit on hospital mortality and length of stay. BMC Health Serv Res 2008;**8**:259.

17. Forster AJ, Worthington JR, Hawken S, et al. Using prospective clinical surveillance to identify adverse events in hospital. BMJ Qual Saf 2011;**20**(9):756-63.

18. Wong BM, Dyal S, Etchells EE, et al. Application of a trigger tool in near real time to inform quality improvement activities: a prospective study in a general medicine ward. BMJ Qual Saf 2015;**24**(4):272-81.

19. Lubberding S, Zwaan L, Timmermans DR, et al. The nature and causes of unintended events reported at 10 internal medicine departments. J Patient Saf 2011;**7**(4):224-31.

For peer review only

20. Mason SL, Kuruvilla S, Riga CV, et al. Design and validation of an error capture tool for quality evaluation in the vascular and endovascular surgical theatre. Eur J Vasc Endovasc Surg 2013;**45**(3):248-54.

21. Organisation WH. WHO surgical safety checklist and implementation manual. 2016. http://www.who.int/patientsafety/safesurgery/checklist/en/. Accessed 21st January 2016.

22. Dixon-Woods M, Bosk CL, Aveling EL, et al. Explaining Michigan: developing an ex post theory of a quality improvement program. Milbank Q 2011;**89**(2):167-205.

23. McGinity R, Salokangas M. Introduction: 'embedded research' as an approach into academia for emerging researchers. Management in Education 2014;**28**(1):3-5.

24. Harvey G, Kitson A. PARIHS revisited: from heuristic to integrated framework for the successful implementation of knowledge into practice. Implement Sci 2016;**11**:33.

25. Checkland K, Snow S, McDermott I, et al. 'Animateurs' and animation: what makes a good commissioning manager? Journal of Health Services Research & Policy 2012;**17**(1):11-17.

26. Moore C, Buchanan DA. Sweat the small stuff: A case study of small-scale change processes and consequences in acute care. Health Services Management Research 2013;**26**(1):9-17.

27. Reynolds M, Jheeta S, Benn J, et al. Improving feedback on junior doctors' prescribing errors: mixed-methods evaluation of a quality improvement project. BMJ Qual Saf 2016;10.1136/bmjqs-2015-004717.

For peer review only

28. Pannick S, Beveridge I, Ashrafian H, et al. A stepped wedge, cluster controlled trial of an intervention to improve safety and quality on medical wards: the HEADS-UP study protocol. BMJ Open 2015;**5**(6):e007510.

29. Hemming K, Haines TP, Chilton PJ, et al. The stepped wedge cluster randomised trial: rationale, design, analysis, and reporting. BMJ 2015;**350**.

30. Bion J, Richardson A, Hibbert P, et al. 'Matching Michigan': a 2-year stepped interventional programme to minimise central venous catheter-blood stream infections in intensive care units in England. BMJ Qual Saf 2013;**22**(2):110-23.

31. Health and Social Care Information Centre. Introduction to Healthcare Resource Groups. http://www.hscic.gov.uk/hrg. Accessed 4th February 2016.

32. Sexton JB, Helmreich RL, Neilands TB, et al. The Safety Attitudes Questionnaire: psychometric properties, benchmarking data, and emerging research. BMC Health Serv Res 2006;**6**:44.

33. Havyer RD, Wingo MT, Comfere NI, et al. Teamwork assessment in internal medicine: a systematic review of validity evidence and outcomes. J Gen Intern Med 2014;**29**(6):894-910.

34. Martin J, Taljaard M, Girling A, et al. Systematic review finds major deficiencies in sample size methodology and reporting for stepped-wedge cluster randomised trials. BMJ Open 2016;**6**(2):e010166.

35. Pearse R. Enhanced peri-operative care for high-risk patients (EPOCH) trial: a stepped wedge cluster randomised trial of a quality improvement intervention for patients undergoing emergency laparotomy. http://www.epochtrial.org/docs/EPOCH Protocol revision V2 0 280414.pdf. Accessed 10th December 2014.

For peer review only

36. Hemming K, Taljaard M. Sample size calculations for stepped wedge and cluster randomised trials: a unified approach. J Clin Epidemiol 2016;**69**:137-46.

37. Health and Social Care Information Centre. Indicator Specification: Summary Hospital-level Mortality Indicator methodology (Appendix D, version 1.17). http://www.hscic.gov.uk/media/11151/Indicator-Specification-Summary-Hospital-level-Mortality-Indicator-methodology/pdf/SHMI_Specification.pdf. Accessed 9th October 2014.

38. NASA-Task Load Index (NASA-TLX); 20 Years Later. Proceedings of the Human Factors and Ergonomics Society 50th Annual Meeting; 2006; Santa Monica. HFES.

39. Mitchell I, Schuster A, Smith K, et al. Patient safety incident reporting: a qualitative study of thoughts and perceptions of experts 15 years after 'To Err is Human'. BMJ Qual Saf 2016;**25**(2):92-9.

40. Hutchinson A, Young TA, Cooper KL, et al. Trends in healthcare incident reporting and relationship to safety and quality data in acute hospitals: results from the National Reporting and Learning System. Qual Saf Health Care 2009;**18**(1):5-10.

41. Anthony T, Murray BW, Sum-Ping JT, et al. Evaluating an evidence-based bundle for preventing surgical site infection: A randomized trial. Arch Surg 2011;**146**(3):263-69.

42. Nicholas LH, Dimick JB. Too much of a good thing? Multi-tasking and distraction in quality improvement. Arch Surg 2011;**146**(3):270.

For peer review only

43. Needleman J, Buerhaus P, Pankratz VS, et al. Nurse staffing and inpatient hospital mortality. N Engl J Med 2011;**364**(11):1037-45.

44. McMullen H, Griffiths C, Leber W, et al. Explaining high and low performers in complex intervention trials: a new model based on diffusion of innovations theory. Trials 2015;**16**:242.

45. Ko HC, Turner TJ, Finnigan MA. Systematic review of safety checklists for use by medical care teams in acute hospital settings--limited evidence of effectiveness. BMC Health Serv Res 2011;**11**:211.

46. Deneckere S, Robyns N, Vanhaecht K, et al. Indicators for Follow-Up of Multidisciplinary Teamwork in Care Processes: Results of an International Expert Panel. Eval Health Prof 2011;**34**(3):258-77.

47. Lemieux-Charles L, McGuire WL. What Do We Know about Health Care Team Effectiveness? A Review of the Literature. Medical Care Research and Review 2006;**63**(3):263-300.

48. McIntosh N, Meterko M, Burgess JF, Jr., et al. Organizational predictors of coordination in inpatient medicine. Health Care Manage Rev 2014;**39**(4):279-92.

49. Health Foundation. Research Scan: Does improving patient safety culture affect patient outcomes? London: 2011.

50. Pannick S, Sevdalis N, Athanasiou T. Beyond clinical engagement: a pragmatic model for quality improvement interventions, aligning clinical and managerial priorities. BMJ Qual Saf 2016;**25**:716-25.

51. Mayer EK, Sevdalis N, Rout S, et al. Surgical Checklist Implementation Project: The Impact of Variable WHO Checklist Compliance on Risk-adjusted Clinical

For peer review only

Outcomes After National Implementation: A Longitudinal Study. Ann Surg 2016;**263**(1):58-63.

52. O'Leary KJ, Killarney A, Hansen LO, et al. Effect of patient-centred bedside rounds on hospitalised patients' decision control, activation and satisfaction with care. BMJ Qual Saf 2015;10.1136/bmjqs-2015-004561.

53. Vincent C, Amalberti R. *Safer Healthcare: Strategies for the Real World.* 1st ed: SpringerOpen; 2016.

54. Zheng S, Tucker AL, Ren ZJ, et al. The Impact of Internal Service Quality on Nurse Inefficiency and Medical Errors. 2016. 10.2139/ssrn.2784503. http://ssrn.com/abstract=2784503. Accessed 11th July 2016.

55. Loudon K, Treweek S, Sullivan F, et al. The PRECIS-2 tool: designing trials that are fit for purpose. BMJ 2015;**350**:h2147.

56. SteelFisher GK, Martin LA, Dowal SL, et al. Learning from the closure of clinical programs: a case series from the Hospital Elder Life Program. J Am Geriatr Soc 2013;**61**(6):999-1004.

57. Cowen S, Ellison SLR. Reporting measurement uncertainty and coverage intervals near natural limits. Analyst 2006;**131**(6):710-17.

LEGENDS FOR FIGURES

Figure 1: Relationship between engagement and frequency of HEADS-UP briefings, and the probability of excess length of stay

Online Figure 1: HEADS-UP briefing pro forma

Online Figure 2: Patient flow diagram

For peer review only

For peer review only

Table 1. Institution characteristics.

	Hospital 1	Hospital 2
Institution type (inpatient admissions / year)		
	Community hospital (52,000)	Academic teaching hospital (186,000)
Participating wards (n)		
	Acute medical unit (2)<br>Gastroenterology/ internal medicine (1)<br>Heart failure/ internal medicine (1)<br>Geriatrics (1)<br>Respiratory / internal medicine (1)	Geriatrics (1)
Existing interdisciplinary practice and approaches to adverse event detection & monitoring		
	Daily interdisciplinary 'board round', typically focused on patients' discharge requirements	Daily interdisciplinary 'board round', typically focused on patients' discharge requirements
	Online incident reporting system	Online incident reporting system
	Unstructured mortality casenote reviews	
Major organisational changes during the study period		
	Incipient institutional merger	Deployment of a new electronic health record

For peer review only

Table 2: Ward and admission characteristics during the intervention period. Values are median (interquartile range) unless otherwise stated.

	Control group	Intervention group
Ward characteristics		
Number of patient admissions	4927	6518
Ward admissions per month	64 (55-153)	77 (64-133)
Length of stay for study patients, days	2.0 (0.8-7.6)	2.2 (0.9-7.3)
Admission characteristics		
Age, years	67 (49-80)	65 (47-78)
Age ≥ 65 years [n, (%)]	2707 (54.9)	3275 (50.2)
Female [n, (%)]	2537 (51.5)	3391 (52.0)
Charlson comorbidity index	3 (0-10)	3 (0-8)
Palliative coding [n, (%)]	50 (1.0)	94 (1.4)

For peer review only

Table 3. Summary of analysis for primary and secondary outcomes.

Outcome	HEADS-UP	Control	Adjusted outcome ratio[§]					
			Intention-to-treat		Moderate fidelity[±]		High fidelity[±±]	
			Outcome ratio (95% CI)	*P value*	*Outcome ratio (95% CI)*	*P value*	*Outcome ratio (95% CI)*	*P value*
Primary outcome								
eLOS, no. (%)	1,714 (26.3)	1279 (26.0)	**1.34 (1.10-1.64)**	**0.004**	0.96 (0.77-1.19)	0.700	**0.78 (0.66-0.94)**	**0.007**
Secondary outcomes								
Readmission within 30 days, no. (%)	950 (14.6)	565 (11.5)	1.11 (0.87-1.41)	0.391	1.00 (0.78-1.29)	0.975	1.09 (0.90-1.33)	0.369
In-hospital death or death/readmission within 30 days, no. (%)	1657 (24.2)	1190 (23.0)	1.00 (0.82-1.22)	0.979	1.07 (0.86-1.31)	0.555	1.09 (0.93-1.29)	0.285
Escalation events/month, median (IQR)[*]	8 (3.5-16)	3 (1-6)	0.83 (0.62-1.10)	0.199	**1.35 (1.04-1.75)**	**0.024**	1.09 (0.88-1.35)	0.440
Complications of care/month, median (IQR)	1 (0-2)	1 (0-3)	0.87 (0.51-1.48)	0.601	1.20 (0.62-2.31)	0.588	1.33 (0.78-2.26)	0.299
Total incident reports/month, median (IQR)	16 (10-23.5)	15.5 (9.25-20)	0.91 (0.76-1.09)	0.305	1.16 (0.95-1.42)	0.148	**1.33 (1.15-1.55)**	**<0.001**
High yield incident reports/month, median (IQR) [**]	11 (6-17.5)	9 (5-14.75)	0.91 (0.73-1.13)	0.400	1.02 (0.81-1.30)	0.838	**1.41 (1.18-1.69)**	**<0.001**
Non-nurse incident reports/month,	2 (0.5-4)	1 (1-2.75)	1.04	0.878	0.61	0.148	**1.60**	**0.019**

median (IQR)	(0.62-1.74)	(0.32-1.19)	**(1.08-2.36)**

§ - Odds ratio for binary outcomes; incidence rate ratio for continuous outcomes

± - moderate fidelity = medium frequency (50-75% working days) and monthly reporting ≥ median issues

±± - high fidelity = high frequency (≥75% working days) and monthly reporting ≥ median issues

* - Escalation events = emergency calls, ITU transfers & referrals to the ICU outreach service

** - High yield incident reports = not relating to slips, trips or falls

For peer review only

Table 4: Effect of PCTS on safety and teamwork attitudes

		PCTS		*P value*
		**Participants**	**Non-participants**	
Mean safety score (SD)		68.4 (13.6)	65.9 (16.9)	0.667
Mean teamwork score (SD)		80.2 (12.1)	79.6 (12.3)	0.524
Estimated marginal mean safety score [95% CI]				
Hospital 1	Baseline	59.8 [51.4-68.1]	69.2 [64.6-73.9]	**0.021***
	6 months	70.3 [65.3-75.2]	61.0 [50.8-71.2]	
Hospital 2	Baseline	70.8 [51.1-90.4]	54.9 [46.2-63.7]	
	6 months	71.0 [56.0-86.0]	75.1 [63.5-86.7]	
Estimated marginal mean teamwork score [95% CI]				
Hospital 1	Baseline	72.3 [65.3-79.2]	82.6 [78.8-86.5]	**0.004***
	6 months	81.7 [77.6-85.8]	70.0 [61.6-78.4]	
Hospital 2	Baseline	84.8 [68.5-100.0**]	74.5 [67.2-81.7]	
	6 months	88.2 [75.7-100.0**]	83.3 [73.7-92.9]	

* *p* value for model effect of interaction term PCTS participation*hospital*time

** The upper bound of the 95% CI for estimated marginal mean climate scores was truncated at 100, the limit of the scale.[57]

For peer review only

Online Table: survey respondents and completion rates

Variable	Baseline	6 months
Professional background [n (%)]		
Consultant physician	8 (13.1)	5 (9.3)
Junior physician	28 (45.9)	35 (64.8)
Senior nurse	15 (24.6)	7 (13.0)
No answer	10 (16.4)	7 (13.0)
Median time on the ward [months]		
	4-6	0-3
Ward specialty [n (%)]		
Acute medicine	19 (31.1)	10 (18.5)
Gastroenterology & general medicine	13 (21.3)	11 (20.4)
Heart failure & general medicine	4 (6.6)	5 (9.3)
Geriatrics	16 (26.2)	14 (25.9)
Respiratory & general medicine	9 (14.8)	12 (22.2)
No answer	0	2 (3.7)
Participated in more than 5 HEADS-UP briefings [n (%)]		
Yes	13 (21.3)	37 (68.5)
No	47 (77.0)	14 (25.9)
No answer	1 (1.6)	3 (5.6)
Workload perception score (NASA-TLX) [mean (SD)]		
	13.2 (2.7)	12.8 (3.1)
Unscored safety and teamwork items [n (%)]		
	33/1647 (2.0)	37/1458 (2.5)
Unscored NASA-TLX items [n (%)]		
	21/366 (5.7)	(18/324) (5.6)

For peer review only

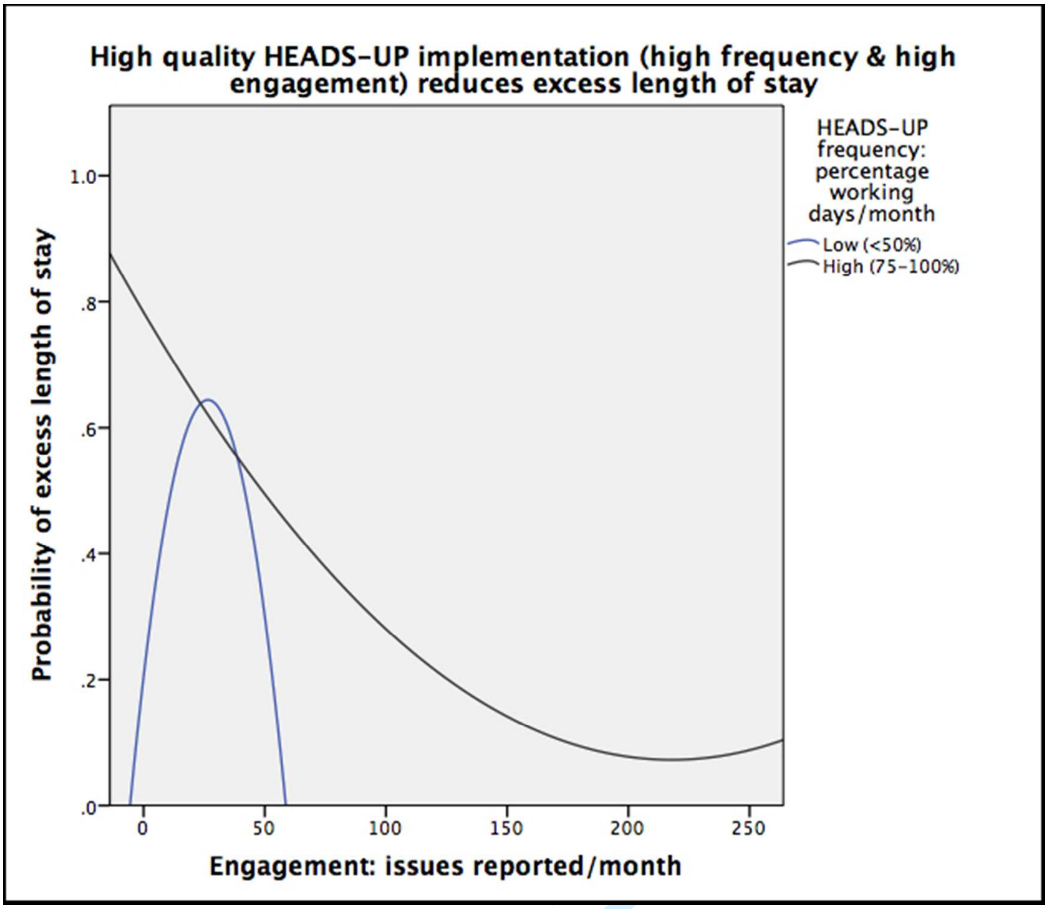

For peer review only

What happened yesterday?

HEADS-UP

A first class hospital for our community

Design by HELIX
helixcentre.com

If a patient has been affected, please include their hospital number. Where there is nothing to report, write N/A in comments box.

1 Review details

Ward:

Scribe name: Role:

Date:

Team members present

- ☐ Clerk
- ☐ OT
- ☐ Discharge team
- ☐ CT
- ☐ HCA
- ☐ Nurse
- ☐ Consultant
- ☐ F2
- ☐ Physio
- ☐ Pharmacist
- ☐ SpR
- ☐ F1

2 Concerns

Staffing levels

- ☐ Medical
- ☐ Nursing
- ☐ OT / PT

Comment or N/A

Equipment

- ☐ Unavailable
- ☐ Not working

Comment or N/A

Services

- ☐ Phlebotomy
- ☐ Echo
- ☐ Radiology
- ☐ Endoscopy
- ☐ In-patient review
  - ☐ Delayed
  - ☐ Too junior

Comment or N/A

Missed results / diagnosis

Comment or N/A

3 Events

Handover / communication problem

Comment or N/A

New hospital-acquired problem

- ☐ Pressure ulcer
- ☐ UTI
- ☐ C-Diff
- ☐ Pneumonia
- ☐ MRSA
- ☐ Other

Comment or N/A

Patient deterioration

- ☐ Sepsis
- ☐ Arrhythmia
- ☐ Delirium
- ☐ Seen by medical team
- ☐ Fall
- ☐ Seen by outreach / ICU

Comment or N/A

Death

- ☐ Expected
- ☐ Unexpected

Comment or N/A

Patient not discharged as expected

Comment or N/A

4 Drug errors and other issues

- ☐ Prescription
- ☐ VTE
- ☐ Antibotics
- ☐ Administration
- ☐ Analgesia
- ☐ Nutrition

Comment or N/A

5 What went really well?

Comment or N/A

6 Who will...

Arrange senior review / outreach for sick patient?	Name or N/A
Change a prescription?	Name or N/A
Escalate equipment problem / discharge delay?	Name or N/A
Complete a DATIX?	Name or N/A
Log patient for M&M discussion?	Name or N/A
Other?	Name or N/A

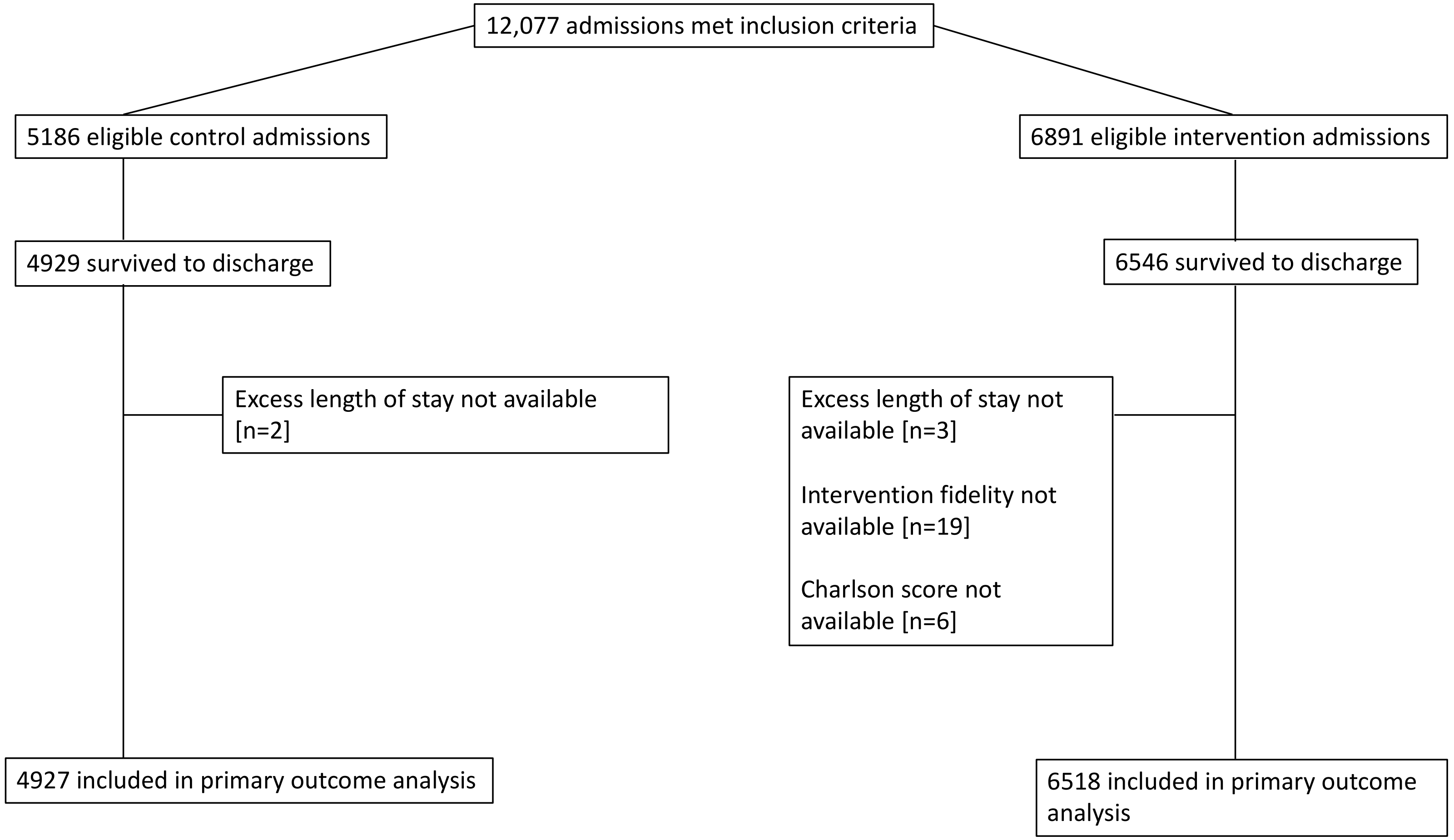
12,077 admissions met inclusion criteria
5186 eligible control admissions
6891 eligible intervention admissions
4929 survived to discharge
6546 survived to discharge
Excess length of stay not available [n=2]
Excess length of stay not available [n=3]
Intervention fidelity not available [n=19]
Charlson score not available [n=6]
4927 included in primary outcome analysis
6518 included in primary outcome analysis

Section/Topic	Item No	Standard Checklist item	Extension for cluster designs	Page No *
**Title and abstract**				
	1a	Identification as a randomised trial in the title	Identification as a cluster randomised trial in the title	1
	1b	Structured summary of trial design, methods, results, and conclusions (for specific guidance see CONSORT for abstracts)[1,2]	See table 2	3
**Introduction**				
**Background and objectives**	2a	Scientific background and explanation of rationale	Rationale for using a cluster design	7
	2b	Specific objectives or hypotheses	Whether objectives pertain to the the cluster level, the individual participant level or both	8
**Methods**				
**Trial design**	3a	Description of trial design (such as parallel, factorial) including allocation ratio	Definition of cluster and description of how the design features apply to the clusters	7
	3b	Important changes to methods after trial commencement (such as eligibility criteria), with reasons		N/A
**Participants**	4a	Eligibility criteria for participants	Eligibility criteria for clusters	7
	4b	Settings and locations where the data were collected		7
**Interventions**	5	The interventions for each group with sufficient details to allow replication, including how and when they were actually administered	Whether interventions pertain to the cluster level, the individual participant level or both	5-7
**Outcomes**	6a	Completely defined pre-specified primary and secondary outcome measures, including how and	Whether outcome measures pertain to the cluster level, the individual participant level or both	8

		when they were assessed		
	6b	Any changes to trial outcomes after the trial commenced, with reasons		N/A
**Sample size**	7a	How sample size was determined	Method of calculation, number of clusters(s) (and whether equal or unequal cluster sizes are assumed), cluster size, a coefficient of intracluster correlation (ICC or *k*), and an indication of its uncertainty	8
	7b	When applicable, explanation of any interim analyses and stopping guidelines		N/A
**Randomisation:**				
**Sequence generation**	8a	Method used to generate the random allocation sequence		7
	8b	Type of randomisation; details of any restriction (such as blocking and block size)	Details of stratification or matching if used	7
**Allocation concealment mechanism**	9	Mechanism used to implement the random allocation sequence (such as sequentially numbered containers), describing any steps taken to conceal the sequence until interventions were assigned	Specification that allocation was based on clusters rather than individuals and whether allocation concealment (if any) was at the cluster level, the individual participant level or both	8
**Implementation**	10	Who generated the random allocation sequence, who enrolled participants, and who assigned participants to interventions	Replace by 10a, 10b and 10c	
	10a		Who generated the random allocation sequence, who enrolled clusters, and who assigned clusters to interventions	7
	10b		Mechanism by which individual participants were included in clusters for the purposes of the	7

For peer review only - http://bmjopen.bmj.com/site/about/guidelines.xhtml

			trial (such as complete enumeration, random sampling)	
	10c		From whom consent was sought (representatives of the cluster, or individual cluster members, or both), and whether consent was sought before or after randomisation	7
**Blinding**	11a	If done, who was blinded after assignment to interventions (for example, participants, care providers, those assessing outcomes) and how		8
	11b	If relevant, description of the similarity of interventions		N/A
**Statistical methods**	12a	Statistical methods used to compare groups for primary and secondary outcomes	How clustering was taken into account	9
	12b	Methods for additional analyses, such as subgroup analyses and adjusted analyses		9
**Results**				
**Participant flow (a diagram is strongly recommended)**	13a	For each group, the numbers of participants who were randomly assigned, received intended treatment, and were analysed for the primary outcome	For each group, the numbers of clusters that were randomly assigned, received intended treatment, and were analysed for the primary outcome	10
	13b	For each group, losses and exclusions after randomisation, together with reasons	For each group, losses and exclusions for both clusters and individual cluster members	Supplement
**Recruitment**	14a	Dates defining the periods of recruitment and follow-up		7
	14b	Why the trial ended or was stopped		7
**Baseline data**	15	A table showing baseline	Baseline characteristics for the	19

For peer review only

		demographic and clinical characteristics for each group	individual and cluster levels as applicable for each group	
**Numbers analysed**	16	For each group, number of participants (denominator) included in each analysis and whether the analysis was by original assigned groups	For each group, number of clusters included in each analysis	Supplement
**Outcomes and estimation**	17a	For each primary and secondary outcome, results for each group, and the estimated effect size and its precision (such as 95% confidence interval)	Results at the individual or cluster level as applicable and a coefficient of intracluster correlation (ICC or k) for each primary outcome	10-11
	17b	For binary outcomes, presentation of both absolute and relative effect sizes is recommended		10-11
**Ancillary analyses**	18	Results of any other analyses performed, including subgroup analyses and adjusted analyses, distinguishing pre-specified from exploratory		10-11
**Harms**	19	All important harms or unintended effects in each group (for specific guidance see CONSORT for harms[3])		N/A
**Discussion**				
**Limitations**	20	Trial limitations, addressing sources of potential bias, imprecision, and, if relevant, multiplicity of analyses		11-13
**Generalisability**	21	Generalisability (external validity, applicability) of the trial findings	Generalisability to clusters and/or individual participants (as relevant)	13
**Interpretation**	22	Interpretation consistent with results, balancing benefits and harms, and considering other relevant evidence		11-13
**Other information**				

**Registration**	23	Registration number and name of trial registry	4
**Protocol**	24	Where the full trial protocol can be accessed, if available	7
**Funding**	25	Sources of funding and other support (such as supply of drugs), role of funders	2

** Note: page numbers optional depending on journal requirements*

For peer review only

For peer review only

BMJ Open

Translating staff experience into organisational improvement: the HEADS-UP stepped wedge cluster controlled trial

Journal:	*BMJ Open*
Manuscript ID	bmjopen-2016-014333.R1
Article Type:	Research
Date Submitted by the Author:	14-Feb-2017

Complete List of Authors:	Pannick, Samuel; Imperial College London, NIHR Imperial Patient Safety Translational Research Centre<br>Athanasiou, Thanos; Imperial College London, Surgery and Cancer<br>Long, Susannah; Imperial College London, Centre for Patient Safety and Service Quality<br>Beveridge, Iain; West Middlesex University Hospital NHS Trust, Department of Medicine<br>Sevdalis, Nick; King's College London,

**Primary Subject Heading**:	Health services research
Secondary Subject Heading:	Medical management
Keywords:	patient safety, Quality in health care < HEALTH SERVICES ADMINISTRATION & MANAGEMENT, stepped wedge, medical ward, prospective clinical surveillance

SCHOLARONE™
Manuscripts

For peer review only

Translating staff experience into organisational improvement: the HEADS-UP stepped wedge cluster controlled trial

Samuel Pannick, MA MBBS, MRCP, PhD[1]; Thanos Athanasiou, MD, PhD[2]; Susannah J Long, MA MB BS, FRCP, PhD[1,3]; Iain Beveridge, MB, BChir, FRCP[4]; and Nick Sevdalis, PhD[5]

[1] NIHR Imperial Patient Safety Translational Research Centre, Imperial College London, London, UK

[2] Department of Surgery & Cancer, Imperial College London, London, UK

[3] Imperial College Healthcare NHS Trust, London, UK

[4] West Middlesex University Hospital NHS Trust, Isleworth, UK

[5] Centre for Implementation Science, King's College London, London, UK

Trial registration: ISRCTN 34806867 (http://www.isrctn.com/ISRCTN34806867)

Corresponding author:

Dr Sam Pannick, MA MB BS MRCP PhD

Room 503 Medical School Building, Norfolk Place, London W2 1PG. United Kingdom

s.pannick@imperial.ac.uk; + (44) 207 3543149

Key words: interdisciplinary care; medical wards; patient safety; stepped wedge trial

Contributions:

Study design: SP, TA, NS. Study implementation: SP, SJL, IB. Statistical analysis: SP, TA. All authors contributed to, read and approved the final manuscript. Dr Pannick had full access to all of the data in the study and takes responsibility for the integrity of the data and the accuracy of the data analysis.

Financial support:

This paper represents independent research supported by the National Institute for Health Research (NIHR) Imperial Patient Safety Translational Research Centre,

For peer review only

Imperial College Healthcare Charity (Grant GG14\1022), and West Middlesex University Hospital NHS Trust. NS' research is supported by the NIHR Collaboration for Leadership in Applied Health Research and Care South London at King's College Hospital NHS Foundation Trust. NS is a member of King's Improvement Science, which is part of the NIHR CLAHRC South London and comprises a specialist team of improvement scientists and senior researchers based at King's College London. Its work is funded by King's Health Partners (Guy's and St Thomas' NHS Foundation Trust, King's College Hospital NHS Foundation Trust, King's College London and South London and Maudsley NHS Foundation Trust), Guy's and St Thomas' Charity, the Maudsley Charity and the Health Foundation. NS is the director of London Training & Safety Solutions Ltd, which delivers team assessment and training to hospitals on a consultancy basis. No funding source had any role in the design and conduct of the study; collection, management, analysis or interpretation of the data; or preparation, review or approval of the manuscript. The views expressed are those of the authors and not necessarily those of the NHS, the NIHR or the Department of Health.

Abstract length: 300 words

Manuscript length: 3,530 words

Tables: 4

Figures: 2

Online content: supplement

For peer review only

ABSTRACT

Objectives:

Frontline insights into care delivery correlate with patients' clinical outcomes. These outcomes might be improved through near-real time identification and mitigation of staff concerns. We evaluated the effects of a prospective frontline surveillance system on patient and team outcomes.

Design:

Prospective, stepped wedge, non-randomised, cluster controlled trial; pre-specified per protocol analysis for high fidelity intervention delivery.

Participants:

Seven interdisciplinary medical ward teams, from two hospitals in the United Kingdom.

Intervention:

Prospective clinical team surveillance (PCTS): structured daily interdisciplinary briefings to capture staff concerns, with organisational facilitation and feedback.

Main measures:

The primary outcome was excess length of stay (eLOS): an admission more than 24 hours longer than the local average for comparable patients. Secondary outcomes included safety and teamwork climates, and incident reporting. Mixed-effects models adjusted for time effects, age, comorbidity, palliation status, and ward admissions. Safety and teamwork climates were measured with the Safety Attitudes Questionnaire. High fidelity PCTS delivery comprised high engagement and high briefing frequency.

Results:

Implementation fidelity was variable, both in briefing frequency (median 78.9% working days/month, interquartile range 30.7%), and engagement (median 70 issues/ward/month, interquartile range 78.5). 1714/6518 (26.3%) intervention

For peer review only

admissions had eLOS *vs* 1279/4927 (26.0%) control admissions, an absolute risk increase of 0.3%. PCTS increased eLOS in the adjusted intention-to-treat model (OR 1.32, 95% CI 1.10-1.58, p=0.003). Conversely, high fidelity PCTS reduced eLOS (OR 0.79, 95% CI 0.67-0.94, p=0.006). High fidelity PCTS also increased total, high yield, and non-nurse incident reports (incidence rate ratios 1.28-1.79, all p<0.002). Sustained PCTS significantly improved safety and teamwork climates over time.

Conclusions:

This study highlighted the potential benefits and pitfalls of ward-level interdisciplinary interventions. Whilst these interventions can improve care delivery in complex, fluid environments, benefiting team and patient outcomes, the manner of their implementation is paramount. Suboptimal implementation may have an unexpectedly negative impact on performance.

For peer review only

STRENGTHS AND LIMITATIONS OF THIS STUDY

This is the first controlled evaluation of a prospective frontline surveillance strategy on general medical wards.

The pragmatic nature of the trial increases the generalisability of its findings.

It can be difficult to disentangle the effects of interdisciplinary interventions from the characteristics of the teams that implement them best.

This was a non-randomised study, adopting a necessarily pragmatic approach to the order in which participating teams introduced the intervention.

Contamination between the groups, whereby PCTS-generated organisational support was directed to those wards in the control period, may have reduced the intervention's measurable effect.

For peer review only

INTRODUCTION

Frontline staff have a particular insight into the safety and quality of inpatient care. Favorable staff perceptions of care correlate with improved clinical outcomes, including the likelihoods of patient survival and harm-free care.[1,2] Yet frontline concerns do not feature in national safety initiatives, nor are they a priority at a local level.[3,4] Whilst healthcare transformation may only come about by changing the operational realities of care delivery,[5] senior leaders remain reluctant to pursue unvetted reports of frontline problems.[6] Better methods are needed to capitalise on frontline experiences and translate them into organisational action.

Prospective clinical surveillance systems can promote the systematic identification of safety concerns from a frontline perspective. Embedded observers, or visiting facilitators, work with frontline staff to form a structured record of their experience of care delivery – and its consequences. Observational studies suggest this is a more engaging route to improvement than traditional incident reporting.[7,8] The appeal of these systems is clear. They may act at multiple organisational levels, (i) motivating and empowering staff to resolve unit-level issues within their control; (ii) recording frontline successes and challenges to build a rich understanding of safety and resilience across the organisation; and (iii) engaging leadership in attending to frontline concerns. Nonetheless, prospective clinical surveillance has not yet been tested as an intervention. Its impact on patient and team outcomes is unknown.

Here, we sought to evaluate prospective clinical *team* surveillance (PCTS), a novel extension of prospective clinical surveillance, on UK medical wards. PCTS combines structured, frontline interdisciplinary briefings with facilitated organisational escalation of the issues they identified, and feedback. PCTS was tested in a pragmatic, cluster controlled trial, assessing its effects on patient outcomes, and staff safety attitudes and behaviours.

For peer review only - http://bmjopen.bmj.com/site/about/guidelines.xhtml

METHODS

Study design and patients

We conducted a prospective, interventional, non-randomised stepped wedge trial on seven medical wards at two NHS hospitals [Table 1]. The trial was described in a published protocol.[9] Interdisciplinary ward teams were assigned to a multifaceted quality improvement intervention. Wards introduced the intervention at staged intervals over the study period, such that (by the end of the trial) all teams had adopted the intervention and contributed both control- and intervention-group data. Stepped wedge designs are increasingly used to evaluate service-level interventions in acute care.[10-12] The stepwise implementation protocol is helpful when simultaneous rollout of the intervention would be impractical for logistical reasons.[13] Baseline data collection began in August 2013, with implementation of the intervention between December 2013 and February 2015.

Medical ward teams with an existing structure for daily interdisciplinary team meetings, and their managers, were invited to take part. All the approached teams agreed to participate. The order in which wards adopted the intervention was pragmatically guided by local constraints. It would have been counter-productive to insist on a fully randomised implementation schedule. Instead, we sought input from senior ward staff and nursing leadership, trying to identify when they felt they could support the intervention's introduction. In practice, personnel and organisational changes meant that the anticipated leadership support could not be guaranteed. Similar pragmatic approaches have been used in other stepped wedge evaluations.[14] A schematic of the ward-level implementation schedule is provided in the online supplement [supplement Figure 1].

Adult patients admitted to participating wards during the study period were eligible. Exclusion criteria were: (1) less than 50% of the hospital admission spent on the specified ward; (2) discharge to a new skilled care facility (not the patient's previous address) or other hospital; (3) multiple ward transfers; (4) admission to the high dependency unit or ICU; (5) elective admission, or direct admission from another hospital; and (6) surgeon-directed care for more than 24 hours of the admission.

For peer review only - http://bmjopen.bmj.com/site/about/guidelines.xhtml

Other studies have used similar criteria to identify the patients whose outcomes might be most affected by service-level interventions.[15] The included patient group made up around 90% of all inpatients on these wards during the study period. Participating teams were teaching teams with attendings (consultants), residents (specialty trainees), and interns (foundation doctors). Interdisciplinary staff also included ward clerks and nurses, who were typically based on a single ward. Doctors were based largely on one ward, but with some patient commitments elsewhere in the hospital. Physiotherapists and occupational therapists worked on multiple wards. Neither managers nor clinical staff had protected time for quality improvement initiatives.

Intervention programme

i) Structured team briefing

The PCTS programme was based on observational studies showing that medical teams can engage in the rapid identification and review of potential adverse events.[7,16-19] We designed a daily interdisciplinary briefing for structured team self-report on medical wards: the HEADS-UP (Hospital Event Analysis Describing Significant Unanticipated Problems) briefing. HEADS-UP briefings identified clinical and administrative challenges of the preceding 24 hours. They centered on the problems identified most commonly in the medical ward setting, as described in a recent, large, observational European study.[20] Structured team self-report captures the majority of errors documented by a trained observer.[21]

HEADS-UP briefings were co-developed with frontline clinical staff between October and November 2013. At the request of clinicians, a pro forma was developed with a visual format similar to the World Health Organisation's surgical safety checklist[22] [supplement Figure 2]. Best practice recommendations for improvement interventions advise a degree of local flexibility:[23] other teams were able to make minor changes to their unit's pro forma, whilst retaining the overall format. This flexibility maintains the 'hard core' of the intervention, and permits a 'softer periphery' to maximise its uptake. Briefings ended with a team agreement on how to resolve, or escalate, the concerns that had been discussed. After a short period of

For peer review only - http://bmjopen.bmj.com/site/about/guidelines.xhtml

supervision by the programme lead (a resident with a 0.75 full time equivalent commitment, working as an `embedded researcher'[24] [S.P.]), the briefing was devolved to the ward team. Teams introduced daily HEADS-UP briefings between Monday and Friday, at the most appropriate time in their usual workflow. There was no protected time for team training. Briefings could be led by any member of the ward team, from ward clerk to therapist to attending physician.

ii) Facilitation

The second component of the intervention was facilitation, advancing the issues raised in the HEADS-UP briefings to bring about tangible unit-level and organisational changes. Facilitation helps to make sense of quality improvement interventions, aligning them with their participants and the surrounding context.[25] It is necessarily opportunistic and malleable, taking advantage of existing organisational levers. Here, it involved (for example) working with frontline teams to identify and document their areas of concern more effectively; championing those concerns in regular meetings with service leaders and safety committees; and following up on subsequent agreed actions when other priorities threatened their resolution. Other studies that have used a facilitator in a similar way have described the role as an '*animateur*', bringing on board people over whom he has no direct managerial authority.[26,27]

iii) Feedback

The third component of the intervention was feedback to participating teams, managers, governance committees, and senior executives. Feedback summarised and disseminated the information collected in the daily briefings, highlighting HEADS-UP performance in each area, common concerns and challenges, and recurrent or unresolved problems that might require additional support. HEADS-UP data were provided on request to service leads, to support their business planning. System changes arising from HEADS-UP were publicised, e.g., in existing departmental meetings, and via email and posters. As much as possible, feedback delivery was timely, focused on solution-finding, signposted to relevant resources,

For peer review only

and adopted a non-judgmental approach.[28] Facilitation and feedback were provided by the programme lead.

Although described separately here, we hypothesised that the intervention components would be interlinked, in that changes arising from the programme's facilitation and feedback would motivate increasing engagement with HEADS-UP briefings, in turn increasing their ability to bring about change. We anticipated that improvements in interdisciplinary team care effectiveness would be brought about by parallel improvements in ward teams' function, and incremental support service improvement in response to their concerns.

Setting

Characteristics of the two participating institutions are provided in Table 1. Most study wards were in a community general hospital in London. Both institutions faced significant challenges during the study period, with significant turnover in senior staff, mounting service pressures and financial restrictions. One hospital was in the process of a merger, and underwent a major inspection by the healthcare regulator (the Care Quality Commission). The other hospital was introducing an electronic health record.

Study outcomes

The primary outcome was excess length of stay (eLOS). eLOS was a binary variable, representing an admission more than 24 hours above the patient's expected length of stay. Benchmarks for expected length of stay were generated using patient-level Healthcare Resource Groups[29] data from a network of four local hospitals. These hospitals were subject to the same community service restrictions and healthcare economy demands as the study sites. The study design therefore evaluated the extent to which intervention and control wards met this local standard.

Secondary outcomes included patient-level measures of (i) readmission within 30 days and (ii) in-hospital death or death/readmission within 30 days. Ward-aggregate measures included (iii) escalation events (referrals to the ICU outreach service,

For peer review only - http://bmjopen.bmj.com/site/about/guidelines.xhtml

emergency calls and ICU transfers); (iv) complications of care (pressure ulcers, C.difficile infections, and MRSA, MSSA and E.coli bacteremias); and (v) incident reporting characteristics. (vi) Safety and teamwork climates were assessed at baseline and six months into the intervention period, using the relevant subscales from the Safety Attitudes Questionnaire.[30,31] Secondary outcomes (and the per protocol analysis described below) were pre-specified.

Data collection

Anonymised patient-level and ward-level outcomes were extracted from routinely-collected data sets. Potential participants in the HEADS-UP briefings were invited to complete anonymous surveys at baseline and six months later, either by submitting responses electronically (via Survey Monkey) or via a paper questionnaire. Overall response rates were calculated using contemporaneous staffing rosters.

Sample size

Power calculations were conducted for the primary outcome (eLOS), using a recommended methodology for stepped wedge trials.[32] As described in detail in the published protocol,[9] we adopted a conservative intraclass correlation coefficient (ICC) of 0.06, based on the ICCs for length of stay and appropriateness of stay in trials of acute care pathways.[33] We iterated calculations based on the wards with the highest and lowest baseline eLOS rates.[9] With a two-sided $p<0.05$, a sample size of 7840 patients was needed to detect a 2-14% absolute risk reduction with a power between 75% and 100%.[9] This approach to sample size calculations and study power has been used for other stepped wedge trials.[34]

Allocation and blinding

Individual patients were not recruited separately to the study, so we did not anticipate significant bias due to lack of allocation concealment.[35] Staff could not be blinded to their ward's assignment, due to the nature of the intervention. Clinical outcome data and escalation of care data were extracted by local administrative staff blinded to the study, as part of their ordinary duties. Hospital peer group data

For peer review only

were generated by the data extraction services *CHKS* (Alcester, UK) and *Dr Foster* (London, UK), also blinded to intervention group.

Statistical analysis

Multi-level mixed-effects models (Stata, v14.2) were used to evaluate the intervention's effect on each patient- and ward-level outcome. This statistical approach accounts for clustering of outcomes within wards, and repeated measurements over time, representing ward-level variance in patient outcomes as a random effect. For the binary patient-level outcomes, binary logistic models were used; for counts data (complications of care and processes of care), we used Poisson loglinear models. Analyses involving eLOS were restricted to those patients who survived to discharge; no patients were excluded because of 'outlier' length of stay. General linear models were used to analyse survey data, using a difference-in-difference approach with an interaction code for time*intervention at each site (SPSS, v22).

In addition to the intention-to-treat analysis, we conducted a per protocol analysis, evaluating the effect of briefing implementation fidelity. Based on observations during the pilot period, we described implementation fidelity each month as the product of briefing frequency and engagement. High fidelity required both high briefing frequency and correspondingly intensive documentation by the ward. High frequency was coded if briefings took place on ≥75% working days that month. Monthly engagement was defined as high if teams documented more than the median number of issues. An interaction term 'frequency*engagement' was incorporated into each model, with a code for high fidelity where both engagement and frequency were high. Note that this per protocol analysis, which calculated different implementation fidelity codes for each ward-month, allowed for varying fidelity by a single ward team over the course of the study.

Patient-level outcomes were adjusted for time effects, age, Charlson comorbidity index,[36] and palliation status, as well as ward admissions. Ward-aggregate outcomes were adjusted for time effects, seasonal trends, the median Charlson score of

For peer review only - http://bmjopen.bmj.com/site/about/guidelines.xhtml

patients on the ward that month, and the rotation of interns between departments. Survey analysis defined HEADS-UP participation as self-reported engagement in five or more briefings, and adjusted for self-reported workload on the NASA Task Load Index scale,[37] as well as hospital site and time period.

Ethics and consent

The Research and Development authority at each participating institution approved the study as a service development initiative, exempting it from formal ethical evaluation. As noted in the study protocol, the Office for Regulatory Compliance at the Imperial College Academic Health Science Centre initially advised that registration with a clinical trial database was not required for a study of this nature.[9] This decision was reviewed at the authors' request, and the study was registered – prior to completion of data collection – in the ISRCTN registry (https://dx.doi.org/10.1186/ISRCTN34806867).[9] Staff were aware that the service development was being formally evaluated. As is routine for this type of intervention, we did not seek participant-level consent.

RESULTS

12,077 eligible admissions took place during the study period, of which 11,445 were included in the primary analysis [Figure 1]. 4,927 admissions were in the control group and 6,518 admissions were in the intervention group. Monthly ward admissions were higher in the intervention months (77 admissions/ward/month *vs* 64 admissions/ward/month), as was unadjusted length of stay (2.25 days *vs* 2.0 days) [Table 2].

Implementation fidelity

Briefing implementation data were available for 71/73 (97.2%) ward months. There was variation in implementation fidelity, both in terms of briefing frequency (median 78.9% working days/month, interquartile range 30.7%), and engagement (median 70 issues/ward/month, interquartile range 78.5). In the intervention group, 3607/6518 (55.3%) admissions were in high fidelity ward months.

For peer review only

Successful implementation at hospital 1 was recognised by the Care Quality Commission (the UK's healthcare quality inspectorate), whose inspectors observed briefings in use and formally reported them as 'outstanding practice', their highest rating. More limited organisational attention at site 2 resulted in slower programme implementation. At that site, PCTS was only embedding into routine practice as the second survey period began.

Primary outcome: excess length of stay (eLOS)

1714/6518 (26.3%) intervention admissions had eLOS *vs* 1279/4927 (26.0%) control admissions, an absolute risk increase of 0.3% [Table 3]. In the unadjusted model, PCTS had no impact on eLOS (OR 1.07, 95% CI 0.96-1.19, p=0.219). When adjusted for patient- and ward-level covariates, the intention-to-treat model showed increasing eLOS with PCTS (OR 1.32, 95% CI 1.10-1.58, p=0.003). Of note, there was an underlying trend of improved performance as the study period progressed (OR 0.98, 95% CI 0.96-0.996, p=0.016). The ICC for eLOS was 0.07 (95% CI 0.018-0.250).

The apparent worsening of performance with PCTS did not reflect whether, or how, the intervention had been used. In the planned per protocol analysis, high fidelity PCTS implementation significantly reduced eLOS: OR 0.79, 95% CI 0.67-0.94, p=0.006. An exploratory plot of the modelled relationship between engagement, briefing frequency and the probability of eLOS highlighted different outcomes with high fidelity implementation and lower fidelity implementation [Figure 2]. The plot models engagement and briefing frequency as continuous variables within a multi-level model to help visualise their interaction.

Secondary outcomes

No changes were seen in readmissions or the composite of deaths/readmissions, irrespective of implementation fidelity (all confidence intervals include unity; all p values >0.2). Escalation events and complications of care were also unchanged [Table 2].

For peer review only

High fidelity implementation increased the total number of incident reports submitted each month (IRR 1.28, 95% CI 1.12-1.47, p<0.001), with increases in non-falls incident reports (IRR 1.40, 95% CI 1.19-1.65, p<0.001) and reports from non-nursing staff (IRR 1.79, 95% CI 1.24-2.58, p=0.002).

Survey response rates were 71.8% (61/85) at baseline and 65.1% (54/83) at six months, with 97.7% safety and teamwork questions completed [Supplement]. Unadjusted mean safety and teamwork scores were non-significantly higher after PCTS participation [Table 4]. With sustained implementation, PCTS improved adjusted safety and teamwork scores over time (site 1 estimated marginal mean [EMM] safety score 70.3 *vs* 61.0, p=0.021; EMM teamwork score 81.7 *vs* 70.0, p=0.004). The significant improvement persisted in a sensitivity analysis that adjusted survey results for ward-level as well as hospital-level clustering. 71.9% (23/32) of site 1 respondents agreed they had reported concerns differently as a result of the programme. Of note, higher workload scores correlated with lower safety scores (B= -1.52, 95% CI -2.59 – -0.44, p=0.006) but not teamwork scores (B= -0.21, 95% CI -1.10 – 0.68, p= 0.64).

DISCUSSION

In this stepped wedge cluster controlled trial, intention-to-treat and per protocol analyses produced conflicting results. eLOS increased overall with PCTS, but was reduced by high fidelity PCTS implementation. Amongst the secondary outcomes, safety and teamwork climates improved with sustained PCTS implementation. High fidelity PCTS increased both incident reporting by non-nursing staff, and the number of non-falls reports.

There are several possible explanations for the tension between the two analyses. First, faithful attention to quality improvement efforts may indeed result in worse outcomes, perhaps by distracting attention from existing good practice.[38] This is unlikely to have been the mechanism here: the wards that dedicated most time and effort to the intervention saw improved outcomes. Second, there may have been unmeasured confounders distinguishing between high fidelity and low fidelity PCTS

For peer review only - http://bmjopen.bmj.com/site/about/guidelines.xhtml

teams. Teams that participate more wholeheartedly in trials may be more innovative, with strong leadership, a readiness for change, and better managerial relations.[39] It is difficult, therefore, to entirely separate the intervention's effects from the characteristics of the teams that implemented it best. However, the study design mitigated for this, allowing for varying implementation fidelity by a single team across the study period. As the design elicited high fidelity ward *months* rather than high fidelity teams, unmeasured team characteristics should therefore hold less influence over the results. It is unlikely that our results are a simple reflection of teams' pre-existing practice or fluctuations in their workload.

The most likely explanation is that the intervention was used differently by high fidelity and low fidelity wards. In the linked qualitative analysis, high fidelity PCTS improved team autonomy in resolving problems, and facilitated managerial resolution where necessary.[40] By contrast, low fidelity teams may have used PCTS simply to record issues with a degree of frustration, without any positive change in their attitudes or behaviours.[40] The differential uptake of improvement interventions by teams that are already performing well is a difficult challenge to resolve.[41] Rather than enacting change for themselves, low fidelity teams perhaps deferred to facilitation and managerial input, believing they would be sufficient to address unit-level problems. Previous quality improvement programmes have also inadvertently blurred lines of responsibility.[42] Whilst imperfect PCTS implementation might be deleterious, our results suggest that interdisciplinary quality and safety interventions – when implemented well – remain a viable route to improvement.

Earlier studies of prospective clinical surveillance did not evaluate an effect on patient outcomes, and so we can only position our work within a broader literature on ward-level interdisciplinary interventions. The variable implementation of these interventions likely contributes to their disputed impact on length of stay.[43,44] In addition, the experience of PCTS implementation mirrored that of other structured team initiatives. Only 62% of mandated interdisciplinary surgical checklists are completed in their entirety.[45] Similarly, structured bedside rounds may be implemented for only 54% of patients, despite co-creation with frontline staff.[46] The

benefits of these interventions are only identified at high fidelity,[45] and so optimising their implementation should remain a focus for further research.

This study's strengths include its pragmatism: the trial had broad eligibility criteria; a typical clinical setting; little additional investment for study recruitment or follow-up; and flexibility with regard to intervention delivery and adherence.[47] Limitations include the difficult balancing act between trial rigor and implementation effectiveness. Attempts to preserve distinct intervention and control groups may have constrained implementation fidelity, limiting a more natural, collaborative spread of practice. Conversely, contamination between the groups may have reduced the intervention's measurable effect: PCTS-generated organisational support (over which we had no control) was not necessarily directed to areas that first raised concerns. Last, there may have been inadequate adjustment even for known confounders like workload. Ward admissions are an imperfect proxy for workload: demands on staff increase as the numbers of admissions, transfers and discharges all increase.[48] Ward-level staffing data are rarely available in this detail in the UK.[49]

CONCLUSIONS

Overall, PCTS increased eLOS for general medical patients. In contrast, high fidelity PCTS reduced eLOS, improved safety and teamwork attitudes, and increased high-yield incident reporting. Our results suggest that interdisciplinary interventions can improve care delivery in complex, fluid environments like medical wards. The manner in which these interventions are implemented – honouring the spirit of the interventions and replicating their proposed mechanism of actions – is vitally important. Suboptimal implementation may have an unexpectedly negative impact on team performance.

For peer review only

ACKNOWLEDGEMENTS

This paper represents independent research supported by the National Institute for Health Research (NIHR) Imperial Patient Safety Translational Research Centre, Imperial College Healthcare Charity (Grant GG14\1022), and West Middlesex University Hospital NHS Trust. NS' research is supported by the NIHR Collaboration for Leadership in Applied Health Research and Care South London at King's College Hospital NHS Foundation Trust. NS is a member of King's Improvement Science, which is part of the NIHR CLAHRC South London and comprises a specialist team of improvement scientists and senior researchers based at King's College London. Its work is funded by King's Health Partners (Guy's and St Thomas' NHS Foundation Trust, King's College Hospital NHS Foundation Trust, King's College London and South London and Maudsley NHS Foundation Trust), Guy's and St Thomas' Charity, the Maudsley Charity and the Health Foundation. No funding source had any role in the design and conduct of the study; collection, management, analysis or interpretation of the data; or preparation, review or approval of the manuscript. The views expressed are those of the authors and not necessarily those of the NHS, the NIHR or the Department of Health. An earlier version of this work was presented at the Society of Hospital Medicine Annual Meeting 2016.

CONFLICTS OF INTEREST

NS is the director of London Training & Safety Solutions Ltd, which delivers team assessment and training to hospitals on a consultancy basis.

For peer review only

REFERENCES

1. Lawton R, O'Hara JK, Sheard L, et al. Can staff and patient perspectives on hospital safety predict harm-free care? An analysis of staff and patient survey data and routinely collected outcomes. BMJ Qual Saf 2015;**24**(6):369-76.

2. Tvedt C, Sjetne IS, Helgeland J, et al. An observational study: associations between nurse-reported hospital characteristics and estimated 30-day survival probabilities. BMJ Qual Saf 2014;**23**(9):757-64.

3. Tucker AL, Singer SJ, Hayes JE, et al. Front-line staff perspectives on opportunities for improving the safety and efficiency of hospital work systems. Health Serv Res 2008;**43**(5 Pt 2):1807-29.

4. Singer SJ, Gaba DM, Falwell A, et al. Patient safety climate in 92 US hospitals: differences by work area and discipline. Med Care 2009;**47**(1):23-31.

5. Bohmer RM. The Hard Work of Health Care Transformation. N Engl J Med 2016;**375**(8):709-11.

6. Martin GP, McKee L, Dixon-Woods M. Beyond metrics? Utilizing 'soft intelligence' for healthcare quality and safety. Soc Sci Med 2015;**142**:19-26.

7. Wong BM, Dyal S, Etchells EE, et al. Application of a trigger tool in near real time to inform quality improvement activities: a prospective study in a general medicine ward. BMJ Qual Saf 2015;**24**(4):272-81.

8. White WA, Kennedy K, Belgum HS, et al. The Well-Defined Pediatric ICU: Active Surveillance Using Nonmedical Personnel to Capture Less Serious Safety Events. Jt Comm J Qual Patient Saf 2015;**41**(12):550-60.

For peer review only

9. Pannick S, Beveridge I, Ashrafian H, et al. A stepped wedge, cluster controlled trial of an intervention to improve safety and quality on medical wards: the HEADS-UP study protocol. BMJ Open 2015;**5**(6):e007510.

10. Kitson AL, Schultz TJ, Long L, et al. The prevention and reduction of weight loss in an acute tertiary care setting: protocol for a pragmatic stepped wedge randomised cluster trial (the PRoWL project). BMC Health Serv Res 2013;**13**:299.

11. Poldervaart JM, Reitsma JB, Koffijberg H, et al. The impact of the HEART risk score in the early assessment of patients with acute chest pain: design of a stepped wedge, cluster randomised trial. BMC Cardiovasc Disord 2013;**13**:77.

12. Fuller C, Michie S, Savage J, et al. The Feedback Intervention Trial (FIT)--improving hand-hygiene compliance in UK healthcare workers: a stepped wedge cluster randomised controlled trial. PLoS One 2012;**7**(10):e41617.

13. Hemming K, Haines TP, Chilton PJ, et al. The stepped wedge cluster randomised trial: rationale, design, analysis, and reporting. BMJ 2015;**350**.

14. Bion J, Richardson A, Hibbert P, et al. 'Matching Michigan': a 2-year stepped interventional programme to minimise central venous catheter-blood stream infections in intensive care units in England. BMJ Qual Saf 2013;**22**(2):110-23.

15. Wald HL, Glasheen JJ, Guerrasio J, et al. Evaluation of a hospitalist-run acute care for the elderly service. J Hosp Med 2011;**6**(6):313-21.

16. Andrews LB, Stocking C, Krizek T, et al. An alternative strategy for studying adverse events in medical care. Lancet 1997;**349**(9048):309-13.

17. Forster AJ, Fung I, Caughey S, et al. Adverse events detected by clinical surveillance on an obstetric service. Obstet Gynecol 2006;**108**(5):1073-83.

For peer review only - http://bmjopen.bmj.com/site/about/guidelines.xhtml

For peer review only

18. Forster AJ, Kyeremanteng K, Hooper J, et al. The impact of adverse events in the intensive care unit on hospital mortality and length of stay. BMC Health Serv Res 2008;**8**:259.

19. Forster AJ, Worthington JR, Hawken S, et al. Using prospective clinical surveillance to identify adverse events in hospital. BMJ Qual Saf 2011;**20**(9):756-63.

20. Lubberding S, Zwaan L, Timmermans DR, et al. The nature and causes of unintended events reported at 10 internal medicine departments. J Patient Saf 2011;**7**(4):224-31.

21. Mason SL, Kuruvilla S, Riga CV, et al. Design and validation of an error capture tool for quality evaluation in the vascular and endovascular surgical theatre. Eur J Vasc Endovasc Surg 2013;**45**(3):248-54.

22. Organisation WH. WHO surgical safety checklist and implementation manual. 2016. http://www.who.int/patientsafety/safesurgery/checklist/en/. Accessed 21st January 2016.

23. Dixon-Woods M, Bosk CL, Aveling EL, et al. Explaining Michigan: developing an ex post theory of a quality improvement program. Milbank Q 2011;**89**(2):167-205.

24. McGinity R, Salokangas M. Introduction: 'embedded research' as an approach into academia for emerging researchers. Management in Education 2014;**28**(1):3-5.

25. Harvey G, Kitson A. PARIHS revisited: from heuristic to integrated framework for the successful implementation of knowledge into practice. Implement Sci 2016;**11**:33.

For peer review only

26. Checkland K, Snow S, McDermott I, et al. 'Animateurs' and animation: what makes a good commissioning manager? Journal of Health Services Research & Policy 2012;**17**(1):11-17.

27. Moore C, Buchanan DA. Sweat the small stuff: A case study of small-scale change processes and consequences in acute care. Health Services Management Research 2013;**26**(1):9-17.

28. Reynolds M, Jheeta S, Benn J, et al. Improving feedback on junior doctors' prescribing errors: mixed-methods evaluation of a quality improvement project. BMJ Qual Saf 2016;10.1136/bmjqs-2015-004717.

29. Health and Social Care Information Centre. Introduction to Healthcare Resource Groups. http://www.hscic.gov.uk/hrg. Accessed 4th February 2016.

30. Sexton JB, Helmreich RL, Neilands TB, et al. The Safety Attitudes Questionnaire: psychometric properties, benchmarking data, and emerging research. BMC Health Serv Res 2006;**6**:44.

31. Havyer RD, Wingo MT, Comfere NI, et al. Teamwork assessment in internal medicine: a systematic review of validity evidence and outcomes. J Gen Intern Med 2014;**29**(6):894-910.

32. Hemming K, Girling A. A menu-driven facility for power and detectable-difference calculations in stepped-wedge cluster-randomized trials. Stata J 2014;**14**(2):363-80.

33. Kul S, Vanhaecht K, Panella M. Intraclass correlation coefficients for cluster randomized trials in care pathways and usual care: hospital treatment for heart failure. BMC Health Serv Res 2014;**14**:84.

For peer review only

34. Pearse R. Enhanced peri-operative care for high-risk patients (EPOCH) trial: a stepped wedge cluster randomised trial of a quality improvement intervention for patients undergoing emergency laparotomy. http://www.epochtrial.org/docs/EPOCH Protocol revision V2 0 280414.pdf. Accessed 10th December 2014.

35. Hemming K, Taljaard M. Sample size calculations for stepped wedge and cluster randomised trials: a unified approach. J Clin Epidemiol 2016;**69**:137-46.

36. Health and Social Care Information Centre. Indicator Specification: Summary Hospital-level Mortality Indicator methodology (Appendix D, version 1.17). http://www.hscic.gov.uk/media/11151/Indicator-Specification-Summary-Hospital-level-Mortality-Indicator-methodology/pdf/SHMI_Specification.pdf. Accessed 9th October 2014.

37. NASA-Task Load Index (NASA-TLX); 20 Years Later. Proceedings of the Human Factors and Ergonomics Society 50th Annual Meeting; 2006; Santa Monica. HFES.

38. Nicholas LH, Dimick JB. Too much of a good thing? Multi-tasking and distraction in quality improvement. Arch Surg 2011;**146**(3):270.

39. McMullen H, Griffiths C, Leber W, et al. Explaining high and low performers in complex intervention trials: a new model based on diffusion of innovations theory. Trials 2015;**16**:242.

40. Pannick S, Archer S, Johnston MJ, et al. Translating concerns into action: a detailed qualitative evaluation of an interdisciplinary intervention on medical wards. BMJ Open [in press] 2017.

41. Rotter T, Kinsman L, James E, et al. Clinical pathways: effects on professional practice, patient outcomes, length of stay and hospital costs. Cochrane Database Syst Rev 2010;10.1002/14651858.CD006632.pub2(3):CD006632.

42. Dixon-Woods M, Martin G, Tarrant C, et al. Safer Clinical Systems: evaluation findings. London: The Health Foundation, 2014.

43. Pannick S, Davis R, Ashrafian H, et al. Effects of Interdisciplinary Team Care Interventions on General Medical Wards: A Systematic Review. JAMA Intern Med 2015;**175**(8):1288-98.

44. Bhamidipati VS, Elliott DJ, Justice EM, et al. Structure and outcomes of interdisciplinary rounds in hospitalized medicine patients: A systematic review and suggested taxonomy. Journal of Hospital Medicine 2016;10.1002/jhm.2575:n/a-n/a.

45. Mayer EK, Sevdalis N, Rout S, et al. Surgical Checklist Implementation Project: The Impact of Variable WHO Checklist Compliance on Risk-adjusted Clinical Outcomes After National Implementation: A Longitudinal Study. Ann Surg 2016;**263**(1):58-63.

46. O'Leary KJ, Killarney A, Hansen LO, et al. Effect of patient-centred bedside rounds on hospitalised patients' decision control, activation and satisfaction with care. BMJ Qual Saf 2015;10.1136/bmjqs-2015-004561.

47. Loudon K, Treweek S, Sullivan F, et al. The PRECIS-2 tool: designing trials that are fit for purpose. BMJ 2015;**350**:h2147.

48. Needleman J, Buerhaus P, Pankratz VS, et al. Nurse staffing and inpatient hospital mortality. N Engl J Med 2011;**364**(11):1037-45.

For peer review only

49. Griffiths P, Ball J, Murrells T, et al. Registered nurse, healthcare support worker, medical staffing levels and mortality in English hospital trusts: a cross-sectional study. BMJ Open 2016;**6**(2):e008751.

50. Cowen S, Ellison SLR. Reporting measurement uncertainty and coverage intervals near natural limits. Analyst 2006;**131**(6):710-17.

For peer review only

LEGENDS FOR FIGURES

Figure 1: Patient flow diagram

Figure 2: Relationship between engagement and frequency of HEADS-UP briefings, and the probability of excess length of stay

Supplement Figure 1: Ward implementation schedule

Supplement Figure 2: HEADS-UP briefing pro forma

Table 1. Institution characteristics.

	Hospital 1	Hospital 2
Institution type (inpatient admissions / year)		
	Community hospital (52,000)	Academic teaching hospital (186,000)
Participating wards (n)		
	Acute medical unit (2)<br>Gastroenterology/ internal medicine (1)<br>Heart failure/ internal medicine (1)<br>Geriatrics (1)<br>Respiratory / internal medicine (1)	Geriatrics (1)
Existing interdisciplinary practice and approaches to adverse event detection & monitoring		
	Daily interdisciplinary 'board round', typically focused on patients' discharge requirements	Daily interdisciplinary 'board round', typically focused on patients' discharge requirements
	Online incident reporting system	Online incident reporting system
	Unstructured mortality case-note reviews	
Major organisational changes during the study period		
	Incipient institutional merger	Deployment of a new electronic health record

For peer review only

Table 2: Ward and admission characteristics during the intervention period. Values are median (interquartile range) unless otherwise stated.

	Control group	Intervention group
Ward characteristics		
Number of patient admissions	4927	6518
Ward admissions per month	64 (55-153)	77 (64-133)
Length of stay for study patients, days	2.0 (0.8-7.6)	2.2 (0.9-7.3)
Admission characteristics		
Age, years	67 (49-80)	65 (47-78)
Age ≥ 65 years [n, (%)]	2707 (54.9)	3275 (50.2)
Female [n, (%)]	2537 (51.5)	3391 (52.0)
Charlson comorbidity index	3 (0-10)	3 (0-8)
Palliative coding [n, (%)]	50 (1.0)	94 (1.4)

review only

Table 3. Summary of analysis for primary and secondary outcomes.

Outcome	HEADS-UP	Control	Adjusted outcome ratio[§]			
			Intention-to-treat		High fidelity[±]	
			Outcome ratio (95% CI)	*P value*	*Outcome ratio (95% CI)*	*P value*
Primary outcome						
eLOS, no. (%)	1714 (26.3)	1279 (26.0)	**1.32 (1.10-1.58)**	**0.003**	**0.79 (0.67-0.94)**	**0.006**
Secondary outcomes						
Readmission within 30 days, no. (%)	950 (14.6)	565 (11.5)	1.12 (0.91-1.37)	0.296	1.11 (0.93-1.32)	0.247
In-hospital death or death/readmission within 30 days, no. (%)	1657 (24.2)	1190 (23.0)	1.03 (0.86-1.22)	0.759	1.07 (0.92-1.25)	0.363
Escalation events/month, median (IQR)[*]	8 (3.5-16)	3 (1-6)	0.95 (0.74-1.23)	0.713	1.01 (0.83-1.24)	0.919
Complications of care/month, median (IQR)	1 (0-2)	1 (0-3)	0.92 (0.56-1.50)	0.727	1.28 (0.76-2.13)	0.351
Total incident reports/month, median (IQR)	16 (10-23.5)	15.5 (9.25-20)	0.97 (0.82-1.14)	0.673	**1.28 (1.12-1.47)**	**<0.001**
High yield incident reports/month, median (IQR) [**]	11 (6-17.5)	9 (5-14.75)	0.92 (0.75-1.12)	0.400	**1.40 (1.19-1.65)**	**<0.001**
Non-nurse incident reports/month, median (IQR)	2 (0.5-4)	1 (1-2.75)	0.89 (0.55-1.42)	0.618	**1.79 (1.24-2.58)**	**0.002**

For peer review only

§ - Odds ratio for binary outcomes; incidence rate ratio for continuous outcomes

± - high fidelity = high frequency (≥75% working days) and monthly reporting ≥ median issues

* - Escalation events = emergency calls, ITU transfers & referrals to the ICU outreach service

** - High yield incident reports = not relating to slips, trips or falls

NB Patient-level outcomes were adjusted for time effects, age, Charlson comorbidity index, and palliation status, as well as ward admissions. Ward-aggregate outcomes were adjusted for time effects, seasonal trends, the median Charlson score of patients on the ward that month, and the rotation of interns between departments.

For peer review only

Table 4: Effect of PCTS on safety and teamwork attitudes

		PCTS		*P value*
		**Participants**	**Non-participants**	
Mean safety score (SD)				
		68.4 (13.6)	65.9 (16.9)	0.667
Mean teamwork score (SD)				
		80.2 (12.1)	79.6 (12.3)	0.524
Estimated marginal mean safety score [95% CI]				
Hospital 1	Baseline	59.8 [51.4-68.1]	69.2 [64.6-73.9]	**0.021***
	6 months	70.3 [65.3-75.2]	61.0 [50.8-71.2]	
Hospital 2	Baseline	70.8 [51.1-90.4]	54.9 [46.2-63.7]	
	6 months	71.0 [56.0-86.0]	75.1 [63.5-86.7]	
Estimated marginal mean teamwork score [95% CI]				
Hospital 1	Baseline	72.3 [65.3-79.2]	82.6 [78.8-86.5]	**0.004***
	6 months	81.7 [77.6-85.8]	70.0 [61.6-78.4]	
Hospital 2	Baseline	84.8 [68.5-100.0**]	74.5 [67.2-81.7]	
	6 months	88.2 [75.7-100.0**]	83.3 [73.7-92.9]	

* *p* value for model effect of interaction term PCTS participation*hospital*time

** The upper bound of the 95% CI for estimated marginal mean climate scores was truncated at 100, the limit of the scale.[50]

For peer review only

For peer review only

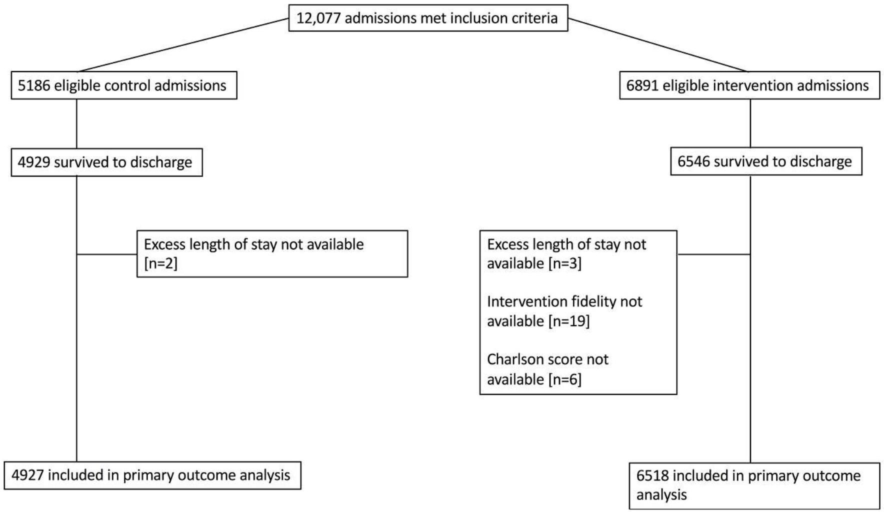

Figure 1: Patient flow diagram

209x148mm (300 x 300 DPI)

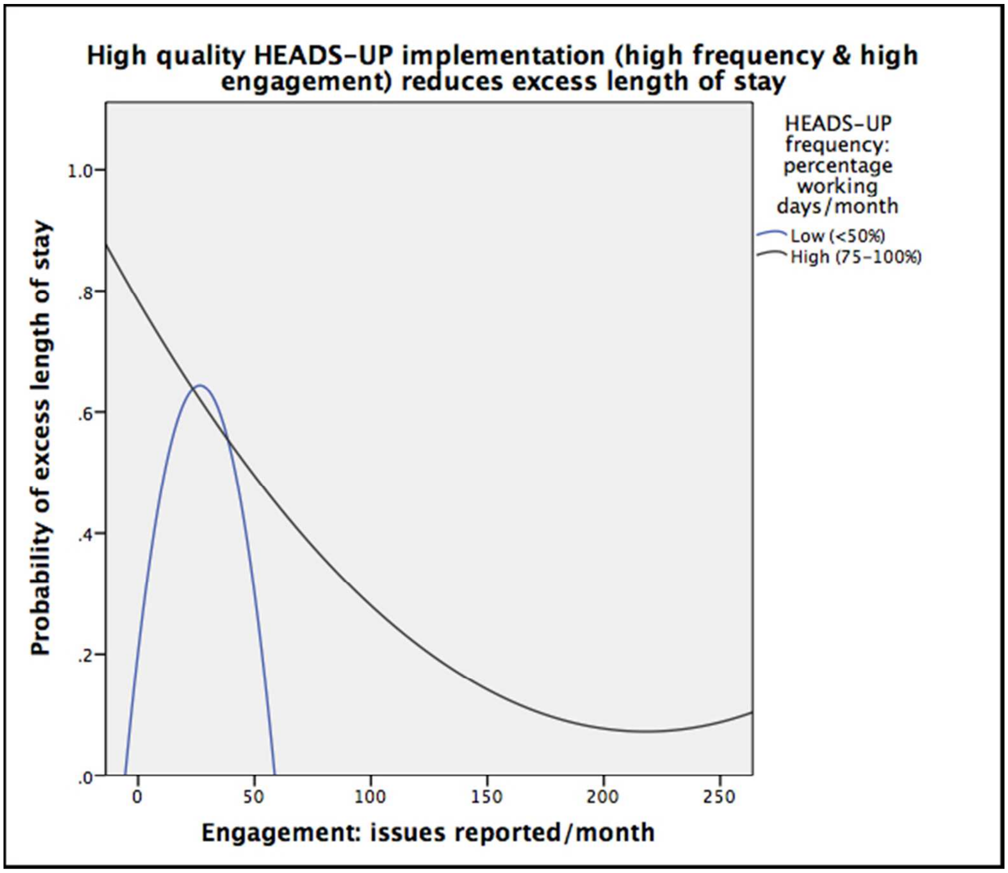

Figure 2: Relationship between engagement and frequency of HEADS-UP briefings, and the probability of excess length of stay

221x191mm (72 x 72 DPI)

only

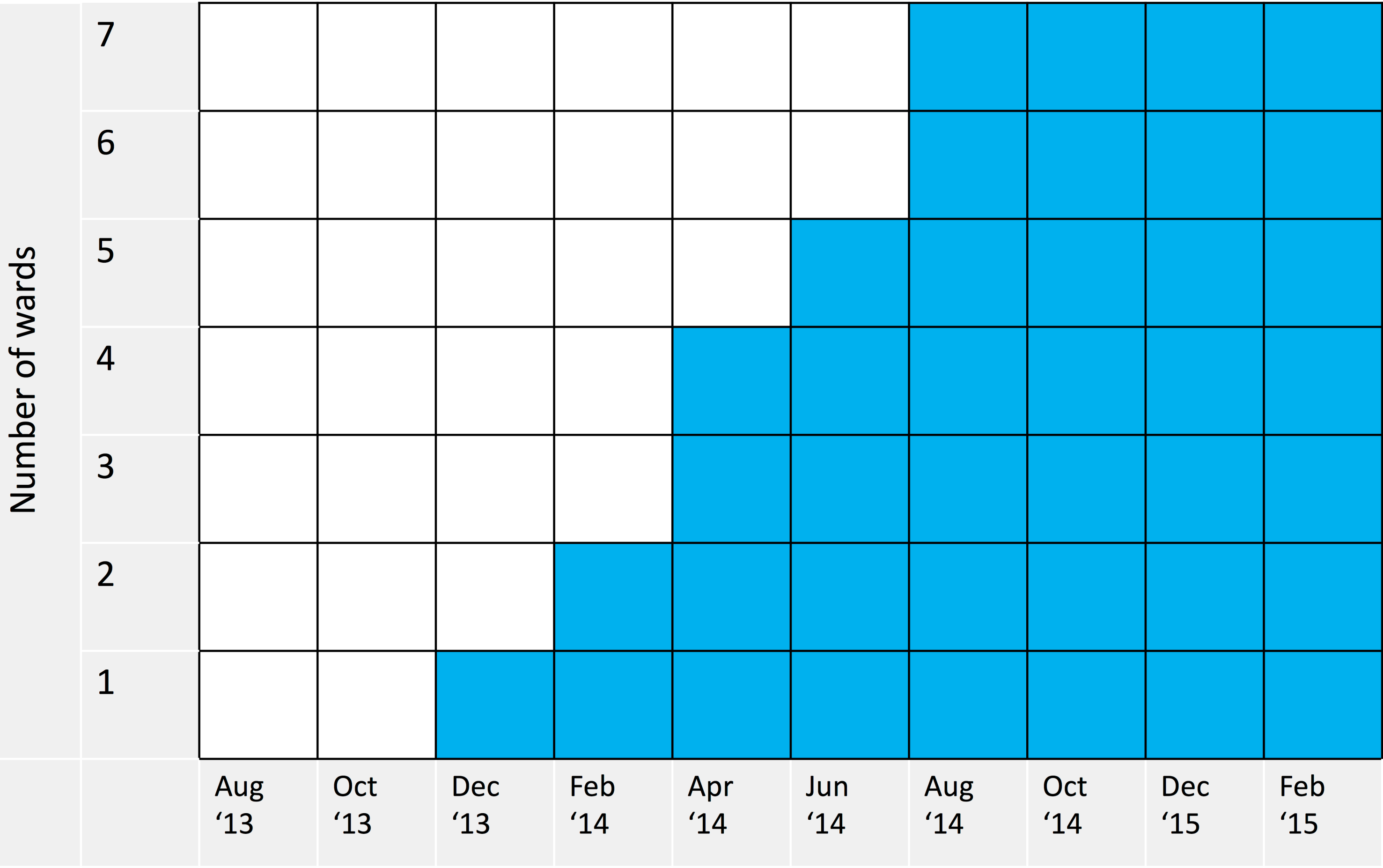
Number of wards
7
6
5
4
3
2
1
Aug ‘13
Oct ‘13
Dec ‘13
Feb ‘14
Apr ‘14
Jun ‘14
Aug ‘14
Oct ‘14
Dec ‘15
Feb ‘15

What happened yesterday?

HEADS-UP

A first class hospital for our community

Design by HELIX
helixcentre.com

If a patient has been affected, please include their hospital number. Where there is nothing to report, write N/A in comments box.

1 Review details

Ward:

Scribe name: Role:

Date:

Team members present

- Clerk
- HCA
- Physio
- OT
- Nurse
- Pharmacist
- Discharge team
- Consultant
- SpR
- CT
- F2
- F1

2 Concerns

Staffing levels

- Medical
- Nursing
- OT / PT

Comment or N/A

Equipment

- Unavailable
- Not working

Comment or N/A

Services

- Phlebotomy
- Echo
- Radiology
- Endoscopy
- In-patient review
  - Delayed
  - Too junior

Comment or N/A

Missed results / diagnosis

Comment or N/A

3 Events

Handover / communication problem

Comment or N/A

New hospital-acquired problem

- Pressure ulcer
- Pneumonia
- UTI
- MRSA
- C-Diff
- Other

Comment or N/A

Patient deterioration

- Sepsis
- Delirium
- Fall
- Arrhythmia
- Seen by medical team
- Seen by outreach / ICU

Comment or N/A

Death

- Expected
- Unexpected

Comment or N/A

Patient not discharged as expected

Comment or N/A

4 Drug errors and other issues

- Prescription
- Administration
- VTE
- Analgesia
- Antibiotics
- Nutrition

Comment or N/A

5 What went really well?

Comment or N/A

6 Who will...

Arrange senior review / outreach for sick patient?	Name or N/A
Change a prescription?	Name or N/A
Escalate equipment problem / discharge delay?	Name or N/A
Complete a DATIX?	Name or N/A
Log patient for M&M discussion?	Name or N/A
Other?	Name or N/A

Supplement Table 1: survey respondents and completion rates

Variable	Baseline	6 months
Professional background [n (%)]		
Consultant physician	8 (13.1)	5 (9.3)
Junior physician	28 (45.9)	35 (64.8)
Senior nurse	15 (24.6)	7 (13.0)
No answer	10 (16.4)	7 (13.0)
Median time on the ward [months]		
	4-6	0-3
Ward specialty [n (%)]		
Acute medicine	19 (31.1)	10 (18.5)
Gastroenterology & general medicine	13 (21.3)	11 (20.4)
Heart failure & general medicine	4 (6.6)	5 (9.3)
Geriatrics	16 (26.2)	14 (25.9)
Respiratory & general medicine	9 (14.8)	12 (22.2)
No answer	0	2 (3.7)
Participated in more than 5 HEADS-UP briefings [n (%)]		
Yes	13 (21.3)	37 (68.5)
No	47 (77.0)	14 (25.9)
No answer	1 (1.6)	3 (5.6)
Workload perception score (NASA-TLX) [mean (SD)]		
	13.2 (2.7)	12.8 (3.1)
Unscored safety and teamwork items [n (%)]		
	33/1647 (2.0)	37/1458 (2.5)
Unscored NASA-TLX items [n (%)]		
	21/366 (5.7)	(18/324) (5.6)

For peer review only

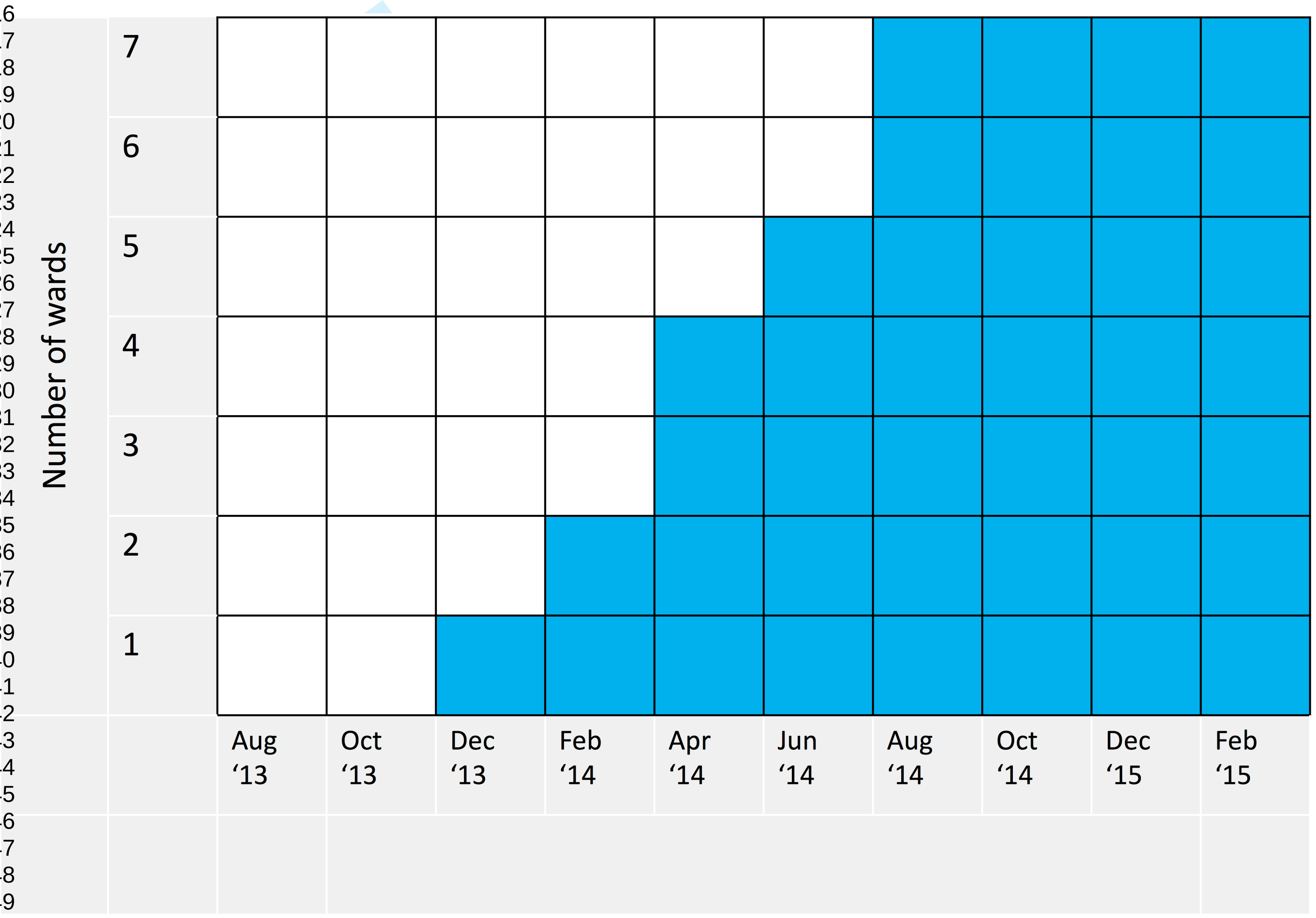
Number of wards
7
6
5
4
3
2
1
Aug '13
Oct '13
Dec '13
Feb '14
Apr '14
Jun '14
Aug '14
Oct '14
Dec '15
Feb '15

Section/Topic	Item No	Standard Checklist item	Extension for cluster designs	Page No *
**Title and abstract**				
	1a	Identification as a randomised trial in the title	Identification as a cluster randomised trial in the title	1
	1b	Structured summary of trial design, methods, results, and conclusions (for specific guidance see CONSORT for abstracts)[1,2]	See table 2	3
**Introduction**				
**Background and objectives**	2a	Scientific background and explanation of rationale	Rationale for using a cluster design	7
	2b	Specific objectives or hypotheses	Whether objectives pertain to the the cluster level, the individual participant level or both	8
**Methods**				
**Trial design**	3a	Description of trial design (such as parallel, factorial) including allocation ratio	Definition of cluster and description of how the design features apply to the clusters	7
	3b	Important changes to methods after trial commencement (such as eligibility criteria), with reasons		N/A
**Participants**	4a	Eligibility criteria for participants	Eligibility criteria for clusters	7
	4b	Settings and locations where the data were collected		7
**Interventions**	5	The interventions for each group with sufficient details to allow replication, including how and when they were actually administered	Whether interventions pertain to the cluster level, the individual participant level or both	5-7
**Outcomes**	6a	Completely defined pre-specified primary and secondary outcome measures, including how and	Whether outcome measures pertain to the cluster level, the individual participant level or both	8

		when they were assessed		
	6b	Any changes to trial outcomes after the trial commenced, with reasons		N/A
**Sample size**	7a	How sample size was determined	Method of calculation, number of clusters(s) (and whether equal or unequal cluster sizes are assumed), cluster size, a coefficient of intracluster correlation (ICC or *k*), and an indication of its uncertainty	8
	7b	When applicable, explanation of any interim analyses and stopping guidelines		N/A
**Randomisation:**				
**Sequence generation**	8a	Method used to generate the random allocation sequence		7
	8b	Type of randomisation; details of any restriction (such as blocking and block size)	Details of stratification or matching if used	7
**Allocation concealment mechanism**	9	Mechanism used to implement the random allocation sequence (such as sequentially numbered containers), describing any steps taken to conceal the sequence until interventions were assigned	Specification that allocation was based on clusters rather than individuals and whether allocation concealment (if any) was at the cluster level, the individual participant level or both	8
**Implementation**	10	Who generated the random allocation sequence, who enrolled participants, and who assigned participants to interventions	Replace by 10a, 10b and 10c	
	10a		Who generated the random allocation sequence, who enrolled clusters, and who assigned clusters to interventions	7
	10b		Mechanism by which individual participants were included in clusters for the purposes of the	7

For peer review only

			trial (such as complete enumeration, random sampling)	
	10c		From whom consent was sought (representatives of the cluster, or individual cluster members, or both), and whether consent was sought before or after randomisation	7
**Blinding**	11a	If done, who was blinded after assignment to interventions (for example, participants, care providers, those assessing outcomes) and how		8
	11b	If relevant, description of the similarity of interventions		N/A
**Statistical methods**	12a	Statistical methods used to compare groups for primary and secondary outcomes	How clustering was taken into account	9
	12b	Methods for additional analyses, such as subgroup analyses and adjusted analyses		9
**Results**				
**Participant flow (a diagram is strongly recommended)**	13a	For each group, the numbers of participants who were randomly assigned, received intended treatment, and were analysed for the primary outcome	For each group, the numbers of clusters that were randomly assigned, received intended treatment, and were analysed for the primary outcome	10
	13b	For each group, losses and exclusions after randomisation, together with reasons	For each group, losses and exclusions for both clusters and individual cluster members	Supplement
**Recruitment**	14a	Dates defining the periods of recruitment and follow-up		7
	14b	Why the trial ended or was stopped		7
**Baseline data**	15	A table showing baseline	Baseline characteristics for the	19

For peer review only

		demographic and clinical characteristics for each group	individual and cluster levels as applicable for each group	
**Numbers analysed**	16	For each group, number of participants (denominator) included in each analysis and whether the analysis was by original assigned groups	For each group, number of clusters included in each analysis	Supplement
**Outcomes and estimation**	17a	For each primary and secondary outcome, results for each group, and the estimated effect size and its precision (such as 95% confidence interval)	Results at the individual or cluster level as applicable and a coefficient of intracluster correlation (ICC or k) for each primary outcome	10-11
	17b	For binary outcomes, presentation of both absolute and relative effect sizes is recommended		10-11
**Ancillary analyses**	18	Results of any other analyses performed, including subgroup analyses and adjusted analyses, distinguishing pre-specified from exploratory		10-11
**Harms**	19	All important harms or unintended effects in each group (for specific guidance see CONSORT for harms[3])		N/A
**Discussion**				
**Limitations**	20	Trial limitations, addressing sources of potential bias, imprecision, and, if relevant, multiplicity of analyses		11-13
**Generalisability**	21	Generalisability (external validity, applicability) of the trial findings	Generalisability to clusters and/or individual participants (as relevant)	13
**Interpretation**	22	Interpretation consistent with results, balancing benefits and harms, and considering other relevant evidence		11-13
**Other information**				

For peer review only - http://bmjopen.bmj.com/site/about/guidelines.xhtml

**Registration**	23	Registration number and name of trial registry	4
**Protocol**	24	Where the full trial protocol can be accessed, if available	7
**Funding**	25	Sources of funding and other support (such as supply of drugs), role of funders	2

** Note: page numbers optional depending on journal requirements*

For peer review only

For peer review only

BMJ Open

Translating staff experience into organisational improvement: the HEADS-UP stepped wedge, cluster controlled, non-randomised trial

Journal:	*BMJ Open*
Manuscript ID	bmjopen-2016-014333.R2
Article Type:	Research
Date Submitted by the Author:	29-Mar-2017

Complete List of Authors:	Pannick, Samuel; Imperial College London, NIHR Imperial Patient Safety Translational Research Centre<br>Athanasiou, Thanos; Imperial College London, Surgery and Cancer<br>Long, Susannah; Imperial College London, Centre for Patient Safety and Service Quality<br>Beveridge, Iain; West Middlesex University Hospital NHS Trust, Department of Medicine<br>Sevdalis, Nick; King's College London,

**Primary Subject Heading**:	Health services research
Secondary Subject Heading:	Medical management
Keywords:	patient safety, Quality in health care < HEALTH SERVICES ADMINISTRATION & MANAGEMENT, stepped wedge, medical ward, prospective clinical surveillance

SCHOLARONE™ Manuscripts

For peer review only

Translating staff experience into organisational improvement: the HEADS-UP stepped wedge, cluster controlled, non-randomised trial

Samuel Pannick, MA MBBS, MRCP, PhD[1]; Thanos Athanasiou, MD, PhD[2]; Susannah J Long, MA MB BS, FRCP, PhD[1,3]; Iain Beveridge, MB, BChir, FRCP[4]; and Nick Sevdalis, PhD[5]

[1] NIHR Imperial Patient Safety Translational Research Centre, Imperial College London, London, UK

[2] Department of Surgery & Cancer, Imperial College London, London, UK

[3] Imperial College Healthcare NHS Trust, London, UK

[4] West Middlesex University Hospital NHS Trust, Isleworth, UK

[5] Centre for Implementation Science, King's College London, London, UK

Trial registration: ISRCTN 34806867 (http://www.isrctn.com/ISRCTN34806867)

Corresponding author:

Dr Sam Pannick, MA MB BS MRCP PhD

Room 503 Medical School Building, Norfolk Place, London W2 1PG. United Kingdom

s.pannick@imperial.ac.uk; + (44) 207 3543149

Key words: interdisciplinary care; medical wards; patient safety; stepped wedge trial

Contributions:

Study design: SP, TA, NS. Study implementation: SP, SJL, IB. Statistical analysis: SP, TA. All authors contributed to, read and approved the final manuscript. Dr Pannick had full access to all of the data in the study and takes responsibility for the integrity of the data and the accuracy of the data analysis.

Financial support:

This paper represents independent research supported by the National Institute for Health Research (NIHR) Imperial Patient Safety Translational Research Centre,

For peer review only

Imperial College Healthcare Charity (Grant GG14\1022), and West Middlesex University Hospital NHS Trust. NS' research is supported by the NIHR Collaboration for Leadership in Applied Health Research and Care South London at King's College Hospital NHS Foundation Trust. NS is a member of King's Improvement Science, which is part of the NIHR CLAHRC South London and comprises a specialist team of improvement scientists and senior researchers based at King's College London. Its work is funded by King's Health Partners (Guy's and St Thomas' NHS Foundation Trust, King's College Hospital NHS Foundation Trust, King's College London and South London and Maudsley NHS Foundation Trust), Guy's and St Thomas' Charity, the Maudsley Charity and the Health Foundation. NS is the director of London Training & Safety Solutions Ltd, which delivers team assessment and training to hospitals on a consultancy basis. No funding source had any role in the design and conduct of the study; collection, management, analysis or interpretation of the data; or preparation, review or approval of the manuscript. The views expressed are those of the authors and not necessarily those of the NHS, the NIHR or the Department of Health.

Abstract length: 300 words

Manuscript length: 3,598 words

Tables: 4

Figures: 2

Online content: supplement

For peer review only

ABSTRACT

Objectives:

Frontline insights into care delivery correlate with patients' clinical outcomes. These outcomes might be improved through near-real time identification and mitigation of staff concerns. We evaluated the effects of a prospective frontline surveillance system on patient and team outcomes.

Design:

Prospective, stepped wedge, non-randomised, cluster controlled trial; pre-specified per protocol analysis for high fidelity intervention delivery.

Participants:

Seven interdisciplinary medical ward teams, from two hospitals in the United Kingdom.

Intervention:

Prospective clinical team surveillance (PCTS): structured daily interdisciplinary briefings to capture staff concerns, with organisational facilitation and feedback.

Main measures:

The primary outcome was excess length of stay (eLOS): an admission more than 24 hours longer than the local average for comparable patients. Secondary outcomes included safety and teamwork climates, and incident reporting. Mixed-effects models adjusted for time effects, age, comorbidity, palliation status, and ward admissions. Safety and teamwork climates were measured with the Safety Attitudes Questionnaire. High fidelity PCTS delivery comprised high engagement and high briefing frequency.

Results:

Implementation fidelity was variable, both in briefing frequency (median 80% working days/month, interquartile range 65-90%), and engagement (median 70 issues/ward/month, interquartile range 34-113). 1714/6518 (26.3%) intervention

For peer review only

admissions had eLOS *vs* 1279/4927 (26.0%) control admissions, an absolute risk increase of 0.3%. PCTS increased eLOS in the adjusted intention-to-treat model (OR 1.32, 95% CI 1.10-1.58, p=0.003). Conversely, high fidelity PCTS reduced eLOS (OR 0.79, 95% CI 0.67-0.94, p=0.006). High fidelity PCTS also increased total, high yield, and non-nurse incident reports (incidence rate ratios 1.28-1.79, all p<0.002). Sustained PCTS significantly improved safety and teamwork climates over time.

Conclusions:

This study highlighted the potential benefits and pitfalls of ward-level interdisciplinary interventions. Whilst these interventions can improve care delivery in complex, fluid environments, benefiting team and patient outcomes, the manner of their implementation is paramount. Suboptimal implementation may have an unexpectedly negative impact on performance.

For peer review only

STRENGTHS AND LIMITATIONS OF THIS STUDY

This is the first controlled evaluation of a prospective frontline surveillance strategy on general medical wards.

The pragmatic nature of the trial increases the generalisability of its findings.

It can be difficult to disentangle the effects of interdisciplinary interventions from the characteristics of the teams that implement them best.

This was a non-randomised study, adopting a necessarily pragmatic approach to the order in which participating teams introduced the intervention.

Contamination between the groups, whereby PCTS-generated organisational support was directed to those wards in the control period, may have reduced the intervention's measurable effect.

For peer review only

INTRODUCTION

Frontline staff have a particular insight into the safety and quality of inpatient care. Favorable staff perceptions of care correlate with improved clinical outcomes, including the likelihoods of patient survival and harm-free care.[1,2] Yet frontline concerns do not feature in national safety initiatives, nor are they a priority at a local level.[3,4] Whilst healthcare transformation may only come about by changing the operational realities of care delivery,[5] senior leaders remain reluctant to pursue unvetted reports of frontline problems.[6] Better methods are needed to capitalise on frontline experiences and translate them into organisational action.

Prospective clinical surveillance systems can promote the systematic identification of safety concerns from a frontline perspective. Embedded observers, or visiting facilitators, work with frontline staff to form a structured record of their experience of care delivery – and its consequences. Observational studies suggest this is a more engaging route to improvement than traditional incident reporting.[7,8] The appeal of these systems is clear. They may act at multiple organisational levels, (i) motivating and empowering staff to resolve unit-level issues within their control; (ii) recording frontline successes and challenges to build a rich understanding of safety and resilience across the organisation; and (iii) engaging leadership in attending to frontline concerns. Nonetheless, prospective clinical surveillance has not yet been tested as an intervention. Its impact on patient and team outcomes is unknown.

Here, we sought to evaluate prospective clinical *team* surveillance (PCTS), a novel extension of prospective clinical surveillance, on UK medical wards. PCTS combines structured, frontline interdisciplinary briefings with facilitated organisational escalation of the issues they identified, and feedback. PCTS was tested in a pragmatic, cluster controlled trial, assessing its effects on patient outcomes, and staff safety attitudes and behaviours.

For peer review only

METHODS

Study design and patients

We conducted a prospective, interventional, non-randomised stepped wedge trial on seven medical wards at two NHS hospitals [Table 1]. The trial was described in a published protocol.[9] Interdisciplinary ward teams were assigned to a multifaceted quality improvement intervention. Wards introduced the intervention at staged intervals over the study period, such that (by the end of the trial) all teams had adopted the intervention and contributed both control- and intervention-group data. Stepped wedge designs are increasingly used to evaluate service-level interventions in acute care.[10-12] The stepwise implementation protocol is helpful when simultaneous rollout of the intervention would be impractical for logistical reasons.[13] Baseline data collection began in August 2013, with implementation of the intervention between December 2013 and February 2015.

Medical ward teams with an existing structure for daily interdisciplinary team meetings, and their managers, were invited to take part. All the approached teams agreed to participate. The order in which wards adopted the intervention was pragmatically guided by local constraints. It would have been counter-productive to insist on a fully randomised implementation schedule. Instead, we sought input from senior ward staff and nursing leadership, trying to identify when they felt they could support the intervention's introduction. In practice, personnel and organisational changes meant that the anticipated leadership support could not be guaranteed. Similar pragmatic approaches have been used in other stepped wedge evaluations.[14] A schematic of the ward-level implementation schedule is provided in the online supplement [supplement Figure 1].

Adult patients admitted to participating wards during the study period were eligible. Exclusion criteria were: (1) less than 50% of the hospital admission spent on the specified ward; (2) discharge to a new skilled care facility (not the patient's previous address) or other hospital; (3) multiple ward transfers; (4) admission to the high dependency unit or ICU; (5) elective admission, or direct admission from another hospital; and (6) surgeon-directed care for more than 24 hours of the admission.

For peer review only - http://bmjopen.bmj.com/site/about/guidelines.xhtml

Other studies have used similar criteria to identify the patients whose outcomes might be most affected by service-level interventions.[15] The included patient group made up around 90% of all inpatients on these wards during the study period. Participating teams were teaching teams with attendings (consultants), residents (specialty trainees), and interns (foundation doctors). Interdisciplinary staff also included ward clerks and nurses, who were typically based on a single ward. Doctors were based largely on one ward, but with some patient commitments elsewhere in the hospital. Physiotherapists and occupational therapists worked on multiple wards. Neither managers nor clinical staff had protected time for quality improvement initiatives.

Intervention programme

i) Structured team briefing

The PCTS programme was based on observational studies showing that medical teams can engage in the rapid identification and review of potential adverse events.[7,16-19] We designed a daily interdisciplinary briefing for structured team self-report on medical wards: the HEADS-UP (Hospital Event Analysis Describing Significant Unanticipated Problems) briefing. HEADS-UP briefings identified clinical and administrative challenges of the preceding 24 hours. They centered on the problems identified most commonly in the medical ward setting, as described in a recent, large, observational European study.[20] Structured team self-report captures the majority of errors documented by a trained observer.[21]

HEADS-UP briefings were co-developed with frontline clinical staff between October and November 2013. At the request of clinicians, a pro forma was developed with a visual format similar to the World Health Organisation's surgical safety checklist[22] [supplement Figure 2]. Best practice recommendations for improvement interventions advise a degree of local flexibility:[23] other teams were able to make minor changes to their unit's pro forma, whilst retaining the overall format. This flexibility maintains the 'hard core' of the intervention, and permits a 'softer periphery' to maximise its uptake. Briefings ended with a team agreement on how to resolve, or escalate, the concerns that had been discussed. After a short period of

For peer review only - http://bmjopen.bmj.com/site/about/guidelines.xhtml

supervision by the programme lead (a resident with a 0.75 full time equivalent commitment, working as an `embedded researcher'[24] [S.P.]), the briefing was devolved to the ward team. Teams introduced daily HEADS-UP briefings between Monday and Friday, at the most appropriate time in their usual workflow. There was no protected time for team training. Briefings could be led by any member of the ward team, from ward clerk to therapist to attending physician.

ii) Facilitation

The second component of the intervention was facilitation, advancing the issues raised in the HEADS-UP briefings to bring about tangible unit-level and organisational changes. Facilitation helps to make sense of quality improvement interventions, aligning them with their participants and the surrounding context.[25] It is necessarily opportunistic and malleable, taking advantage of existing organisational levers. Here, it involved (for example) working with frontline teams to identify and document their areas of concern more effectively; championing those concerns in regular meetings with service leaders and safety committees; and following up on subsequent agreed actions when other priorities threatened their resolution. Other studies that have used a facilitator in a similar way have described the role as an '*animateur*', bringing on board people over whom he has no direct managerial authority.[26,27]

iii) Feedback

The third component of the intervention was feedback to participating teams, managers, governance committees, and senior executives. Feedback summarised and disseminated the information collected in the daily briefings, highlighting HEADS-UP performance in each area, common concerns and challenges, and recurrent or unresolved problems that might require additional support. HEADS-UP data were provided on request to service leads, to support their business planning. System changes arising from HEADS-UP were publicised, e.g., in existing departmental meetings, and via email and posters. As much as possible, feedback delivery was timely, focused on solution-finding, signposted to relevant resources,

For peer review only

and adopted a non-judgmental approach.[28] Facilitation and feedback were provided by the programme lead.

Although described separately here, we hypothesised that the intervention components would be interlinked, in that changes arising from the programme's facilitation and feedback would motivate increasing engagement with HEADS-UP briefings, in turn increasing their ability to bring about change. We anticipated that improvements in interdisciplinary team care effectiveness would be brought about by parallel improvements in ward teams' function, and incremental support service improvement in response to their concerns.

Setting

Characteristics of the two participating institutions are provided in Table 1. Most study wards were in a community general hospital in London. Both institutions faced significant challenges during the study period, with significant turnover in senior staff, mounting service pressures and financial restrictions. One hospital was in the process of a merger, and underwent a major inspection by the healthcare regulator (the Care Quality Commission). The other hospital was introducing an electronic health record.

Study outcomes

The primary outcome was excess length of stay (eLOS). eLOS was a binary variable, representing an admission more than 24 hours above the patient's expected length of stay. Benchmarks for expected length of stay were generated using patient-level Healthcare Resource Groups[29] data from a network of four local hospitals. These hospitals were subject to the same community service restrictions and healthcare economy demands as the study sites. The study design therefore evaluated the extent to which intervention and control wards met this local standard.

Secondary outcomes included patient-level measures of (i) readmission within 30 days and (ii) in-hospital death or death/readmission within 30 days. Ward-aggregate measures included (iii) escalation events (referrals to the ICU outreach service,

For peer review only - http://bmjopen.bmj.com/site/about/guidelines.xhtml

emergency calls and ICU transfers); (iv) complications of care (pressure ulcers, C.difficile infections, and MRSA, MSSA and E.coli bacteremias); and (v) incident reporting characteristics. (vi) Safety and teamwork climates were assessed at baseline and six months into the intervention period, using the relevant subscales from the Safety Attitudes Questionnaire.[30,31] Secondary outcomes (and the per protocol analysis described below) were pre-specified.

Data collection

Anonymised patient-level and ward-level outcomes were extracted from routinely-collected data sets. Potential participants in the HEADS-UP briefings were invited to complete anonymous surveys at baseline and six months later, either by submitting responses electronically (via Survey Monkey) or via a paper questionnaire. Overall response rates were calculated using contemporaneous staffing rosters.

Sample size

Power calculations were conducted for the primary outcome (eLOS), using a recommended methodology for stepped wedge trials.[32] As described in detail in the published protocol,[9] we adopted an intraclass correlation coefficient (ICC) of 0.06, based on the ICCs for length of stay and appropriateness of stay in trials of acute care pathways.[33] We iterated calculations based on the wards with the highest and lowest baseline eLOS rates.[9] With a two-sided $p<0.05$, a sample size of 7840 patients was needed to detect a 2-14% absolute risk reduction with a power between 75% and 100%.[9] This approach to sample size calculations and study power has been used for other stepped wedge trials.[34]

Allocation and blinding

Individual patients were not recruited separately to the study, so we did not anticipate significant bias due to lack of allocation concealment.[35] Staff could not be blinded to their ward's assignment, due to the nature of the intervention. Clinical outcome data and escalation of care data were extracted by local administrative staff blinded to the study, as part of their ordinary duties. Hospital peer group data

For peer review only

were generated by the data extraction services *CHKS* (Alcester, UK) and *Dr Foster* (London, UK), also blinded to intervention group.

Statistical analysis

Multi-level mixed-effects models (Stata, v14.2) were used to evaluate the intervention's effect on each patient- and ward-level outcome. This statistical approach accounts for clustering of outcomes within wards, and repeated measurements over time, representing ward-level variance in patient outcomes as a random effect. For the binary patient-level outcomes, binary logistic models were used; for counts data (complications of care and processes of care), we used Poisson loglinear models. Analyses involving eLOS were restricted to those patients who survived to discharge; no patients were excluded because of 'outlier' length of stay. General linear regression models were used to analyse survey data, using a difference-in-differences approach[36] with an interaction code for time*intervention at each site (SPSS, v22). This method evaluated whether changes in survey responses over time differed between PCTS participants and non-participants.

In addition to the intention-to-treat analysis, we conducted a per protocol analysis, evaluating the effect of briefing implementation fidelity. Based on observations during the pilot period, we described implementation fidelity each month as the product of briefing frequency and engagement. High fidelity required both high briefing frequency and correspondingly intensive documentation by the ward. High frequency was coded if briefings took place on ≥75% working days that month. Monthly engagement was defined as high if teams documented more than the median number of issues. An interaction term 'frequency*engagement' was incorporated into each model, with a code for high fidelity where both engagement and frequency were high. Note that this per protocol analysis, which calculated different implementation fidelity codes for each ward-month, allowed for varying fidelity by a single ward team over the course of the study.

Patient-level outcomes were adjusted for time effects, age, Charlson comorbidity index,[37] and palliation status, as well as ward admissions. Time effects were specified

For peer review only - http://bmjopen.bmj.com/site/about/guidelines.xhtml

as a continuous variable coded for each study month. Ward-aggregate outcomes were adjusted for time effects, seasonal trends, the median Charlson score of patients on the ward that month, and the rotation of interns between departments. Survey analysis defined HEADS-UP participation as self-reported engagement in five or more briefings, and adjusted for self-reported workload on the NASA Task Load Index scale,[38] as well as hospital site and time period.

Ethics and consent

The Research and Development authority at each participating institution approved the study as a service development initiative, exempting it from formal ethical evaluation. As noted in the study protocol, the Office for Regulatory Compliance at the Imperial College Academic Health Science Centre initially advised that registration with a clinical trial database was not required for a study of this nature.[9] This decision was reviewed at the authors' request, and the study was registered – prior to completion of data collection – in the ISRCTN registry (https://dx.doi.org/10.1186/ISRCTN34806867).[9] Staff were aware that the service development was being formally evaluated. As is routine for this type of intervention, we did not seek participant-level consent.

RESULTS

12,077 eligible admissions took place during the study period, of which 11,445 were included in the primary analysis [Figure 1]. 4,927 admissions were in the control group and 6,518 admissions were in the intervention group. Monthly ward admissions were higher in the intervention months (77 admissions/ward/month *vs* 64 admissions/ward/month), as was unadjusted length of stay (2.25 days *vs* 2.0 days) [Table 2].

Implementation fidelity

Briefing implementation data were available for 71/73 (97.2%) ward months. There was variation in implementation fidelity, both in terms of briefing frequency (median 80% working days/month, interquartile range 65%-90%), and engagement (median

For peer review only - http://bmjopen.bmj.com/site/about/guidelines.xhtml

70 issues/ward/month, interquartile range 34-113). In the intervention group, 3607/6518 (55.3%) admissions were in high fidelity ward months.

Successful implementation at hospital 1 was recognised by the Care Quality Commission (the UK's healthcare quality inspectorate), whose inspectors observed briefings in use and formally reported them as 'outstanding practice', their highest rating. More limited organisational attention at site 2 resulted in slower programme implementation. At that site, PCTS was only embedding into routine practice as the second survey period began.

Primary outcome: excess length of stay (eLOS)

1714/6518 (26.3%) intervention admissions had eLOS *vs* 1279/4927 (26.0%) control admissions, an absolute risk increase of 0.3% [Table 3]. In the unadjusted model, eLOS did not change significantly with PCTS (OR 1.07, 95% CI 0.96-1.19, p=0.219). When adjusted for patient- and ward-level covariates, the intention-to-treat model showed increasing eLOS with PCTS (OR 1.32, 95% CI 1.10-1.58, p=0.003). Of note, there was an underlying trend of improved performance as the study period progressed (OR 0.98, 95% CI 0.96-0.996, p=0.016). The ICC for eLOS was 0.07 (95% CI 0.018-0.250).

The apparent worsening of performance with PCTS did not reflect whether, or how, the intervention had been used. In the planned per protocol analysis, high fidelity PCTS implementation significantly reduced eLOS: OR 0.79, 95% CI 0.67-0.94, p=0.006. A sensitivity analysis (coding time effects as a categorical variable) confirmed the effect sizes of both intention-to-treat and per protocol analyses, and their statistical significance.

An exploratory plot of the modelled relationship between engagement, briefing frequency and the probability of eLOS highlighted different outcomes with high fidelity implementation and lower fidelity implementation [Figure 2]. The plot models engagement and briefing frequency as continuous variables within a multi-level model to help visualise their interaction.

For peer review only - http://bmjopen.bmj.com/site/about/guidelines.xhtml

Secondary outcomes

No changes were seen in readmissions or the composite of deaths/readmissions, irrespective of implementation fidelity (all confidence intervals include unity; all p values >0.2). Escalation events and complications of care were also unchanged [Table 2].

There was no change in incident reporting in the intention-to-treat analysis. By contrast, high fidelity implementation increased the total number of incident reports submitted each month (IRR 1.28, 95% CI 1.12-1.47, p<0.001), with increases in non-falls incident reports (IRR 1.40, 95% CI 1.19-1.65, p<0.001) and reports from non-nursing staff (IRR 1.79, 95% CI 1.24-2.58, p=0.002).

Survey response rates were 71.8% (61/85) at baseline and 65.1% (54/83) at six months, with 97.7% safety and teamwork questions completed. The respondent demographics are provided in the online supplement [supplement Table 1]. Unadjusted mean safety and teamwork scores were non-significantly higher after PCTS participation [Table 4]. With sustained implementation, PCTS improved adjusted safety and teamwork scores over time (site 1 estimated marginal mean [EMM] safety score 70.3 *vs* 61.0, p=0.021; EMM teamwork score 81.7 *vs* 70.0, p=0.004). The significant improvement persisted in a sensitivity analysis that adjusted survey results for ward-level as well as hospital-level clustering. 71.9% (23/32) of site 1 respondents agreed they had reported concerns differently as a result of the programme. Of note, higher workload scores correlated with lower safety scores (B= -1.52, 95% CI -2.59 – -0.44, p=0.006) but not teamwork scores (B= -0.21, 95% CI -1.10 – 0.68, p= 0.64).

DISCUSSION

In this stepped wedge cluster controlled trial, intention-to-treat and per protocol analyses produced conflicting results. eLOS increased overall with PCTS, but was reduced by high fidelity PCTS implementation. Amongst the secondary outcomes, safety and teamwork climates improved with sustained PCTS implementation. High

For peer review only - http://bmjopen.bmj.com/site/about/guidelines.xhtml

fidelity PCTS increased both incident reporting by non-nursing staff, and the number of non-falls reports.

There are several possible explanations for the tension between the two analyses. First, faithful attention to quality improvement efforts may indeed result in worse outcomes, perhaps by distracting attention from existing good practice.[39] This is unlikely to have been the mechanism here: the wards that dedicated most time and effort to the intervention saw improved outcomes. Second, there may have been unmeasured confounders distinguishing between high fidelity and low fidelity PCTS teams. Teams that participate more wholeheartedly in trials may be more innovative, with strong leadership, a readiness for change, and better managerial relations.[40] It is difficult, therefore, to entirely separate the intervention's effects from the characteristics of the teams that implemented it best. However, the study design mitigated for this, allowing for varying implementation fidelity by a single team across the study period. As the design elicited high fidelity ward *months* rather than high fidelity teams, unmeasured team characteristics should therefore hold less influence over the results. It is unlikely that our results are a simple reflection of teams' pre-existing practice or fluctuations in their workload.

The most likely explanation is that the intervention was used differently by high fidelity and low fidelity wards. In the linked qualitative analysis, high fidelity PCTS improved team autonomy in resolving problems, and facilitated managerial resolution where necessary.[41] By contrast, low fidelity teams may have used PCTS simply to record issues with a degree of frustration, without any positive change in their attitudes or behaviours.[41] The differential uptake of improvement interventions by teams that are already performing well is a difficult challenge to resolve.[42] Rather than enacting change for themselves, low fidelity teams perhaps deferred to facilitation and managerial input, believing they would be sufficient to address unit-level problems. Previous quality improvement programmes have also inadvertently blurred lines of responsibility.[43] Whilst imperfect PCTS implementation might be deleterious, our results suggest that interdisciplinary quality and safety interventions – when implemented well – remain a viable route to improvement.

For peer review only - http://bmjopen.bmj.com/site/about/guidelines.xhtml

Earlier studies of prospective clinical surveillance did not evaluate an effect on patient outcomes, and so we can only position our work within a broader literature on ward-level interdisciplinary interventions. The variable implementation of these interventions likely contributes to their disputed impact on length of stay.[44,45] In addition, the experience of PCTS implementation mirrored that of other structured team initiatives. Only 62% of mandated interdisciplinary surgical checklists are completed in their entirety.[46] Similarly, structured bedside rounds may be implemented for only 54% of patients, despite co-creation with frontline staff.[47] The benefits of these interventions are only identified at high fidelity,[46] and so optimising their implementation should remain a focus for further research.

This study's strengths include its pragmatism: the trial had broad eligibility criteria; a typical clinical setting; little additional investment for study recruitment or follow-up; and flexibility with regard to intervention delivery and adherence.[48] Limitations include the difficult balancing act between trial rigor and implementation effectiveness. Attempts to preserve distinct intervention and control groups may have constrained implementation fidelity, limiting a more natural, collaborative spread of practice. Conversely, contamination between the groups may have reduced the intervention's measurable effect: PCTS-generated organisational support (over which we had no control) was not necessarily directed to areas that first raised concerns. Last, there may have been inadequate adjustment even for known confounders like workload. Ward admissions are an imperfect proxy for workload: demands on staff increase as the numbers of admissions, transfers and discharges all increase.[49] Ward-level staffing data are rarely available in this detail in the UK.[50]

CONCLUSIONS

Overall, PCTS increased eLOS for general medical patients. In contrast, high fidelity PCTS reduced eLOS, improved safety and teamwork attitudes, and increased high-yield incident reporting. Our results suggest that interdisciplinary interventions can improve care delivery in complex, fluid environments like medical wards. The

manner in which these interventions are implemented – honouring the spirit of the interventions and replicating their proposed mechanism of actions – is vitally important. Suboptimal implementation may have an unexpectedly negative impact on team performance.

ACKNOWLEDGEMENTS

This paper represents independent research supported by the National Institute for Health Research (NIHR) Imperial Patient Safety Translational Research Centre, Imperial College Healthcare Charity (Grant GG14\1022), and West Middlesex University Hospital NHS Trust. NS' research is supported by the NIHR Collaboration for Leadership in Applied Health Research and Care South London at King's College Hospital NHS Foundation Trust. NS is a member of King's Improvement Science, which is part of the NIHR CLAHRC South London and comprises a specialist team of improvement scientists and senior researchers based at King's College London. Its work is funded by King's Health Partners (Guy's and St Thomas' NHS Foundation Trust, King's College Hospital NHS Foundation Trust, King's College London and South London and Maudsley NHS Foundation Trust), Guy's and St Thomas' Charity, the Maudsley Charity and the Health Foundation. No funding source had any role in the design and conduct of the study; collection, management, analysis or interpretation of the data; or preparation, review or approval of the manuscript. The views expressed are those of the authors and not necessarily those of the NHS, the NIHR or the Department of Health. An earlier version of this work was presented at the Society of Hospital Medicine Annual Meeting 2016.

CONFLICTS OF INTEREST

For peer review only

NS is the director of London Training & Safety Solutions Ltd, which delivers team assessment and training to hospitals on a consultancy basis.

DATA SHARING

Anonymised data can be requested from the corresponding author.

For peer review only

REFERENCES

1. Lawton R, O'Hara JK, Sheard L, et al. Can staff and patient perspectives on hospital safety predict harm-free care? An analysis of staff and patient survey data and routinely collected outcomes. BMJ Qual Saf 2015;**24**(6):369-76.

2. Tvedt C, Sjetne IS, Helgeland J, et al. An observational study: associations between nurse-reported hospital characteristics and estimated 30-day survival probabilities. BMJ Qual Saf 2014;**23**(9):757-64.

3. Tucker AL, Singer SJ, Hayes JE, et al. Front-line staff perspectives on opportunities for improving the safety and efficiency of hospital work systems. Health Serv Res 2008;**43**(5 Pt 2):1807-29.

4. Singer SJ, Gaba DM, Falwell A, et al. Patient safety climate in 92 US hospitals: differences by work area and discipline. Med Care 2009;**47**(1):23-31.

5. Bohmer RM. The Hard Work of Health Care Transformation. N Engl J Med 2016;**375**(8):709-11.

6. Martin GP, McKee L, Dixon-Woods M. Beyond metrics? Utilizing 'soft intelligence' for healthcare quality and safety. Soc Sci Med 2015;**142**:19-26.

7. Wong BM, Dyal S, Etchells EE, et al. Application of a trigger tool in near real time to inform quality improvement activities: a prospective study in a general medicine ward. BMJ Qual Saf 2015;**24**(4):272-81.

8. White WA, Kennedy K, Belgum HS, et al. The Well-Defined Pediatric ICU: Active Surveillance Using Nonmedical Personnel to Capture Less Serious Safety Events. Jt Comm J Qual Patient Saf 2015;**41**(12):550-60.

For peer review only

9. Pannick S, Beveridge I, Ashrafian H, et al. A stepped wedge, cluster controlled trial of an intervention to improve safety and quality on medical wards: the HEADS-UP study protocol. BMJ Open 2015;**5**(6):e007510.

10. Kitson AL, Schultz TJ, Long L, et al. The prevention and reduction of weight loss in an acute tertiary care setting: protocol for a pragmatic stepped wedge randomised cluster trial (the PRoWL project). BMC Health Serv Res 2013;**13**:299.

11. Poldervaart JM, Reitsma JB, Koffijberg H, et al. The impact of the HEART risk score in the early assessment of patients with acute chest pain: design of a stepped wedge, cluster randomised trial. BMC Cardiovasc Disord 2013;**13**:77.

12. Fuller C, Michie S, Savage J, et al. The Feedback Intervention Trial (FIT)--improving hand-hygiene compliance in UK healthcare workers: a stepped wedge cluster randomised controlled trial. PLoS One 2012;**7**(10):e41617.

13. Hemming K, Haines TP, Chilton PJ, et al. The stepped wedge cluster randomised trial: rationale, design, analysis, and reporting. BMJ 2015;**350**.

14. Bion J, Richardson A, Hibbert P, et al. 'Matching Michigan': a 2-year stepped interventional programme to minimise central venous catheter-blood stream infections in intensive care units in England. BMJ Qual Saf 2013;**22**(2):110-23.

15. Wald HL, Glasheen JJ, Guerrasio J, et al. Evaluation of a hospitalist-run acute care for the elderly service. J Hosp Med 2011;**6**(6):313-21.

16. Andrews LB, Stocking C, Krizek T, et al. An alternative strategy for studying adverse events in medical care. Lancet 1997;**349**(9048):309-13.

17. Forster AJ, Fung I, Caughey S, et al. Adverse events detected by clinical surveillance on an obstetric service. Obstet Gynecol 2006;**108**(5):1073-83.

For peer review only

18. Forster AJ, Kyeremanteng K, Hooper J, et al. The impact of adverse events in the intensive care unit on hospital mortality and length of stay. BMC Health Serv Res 2008;**8**:259.

19. Forster AJ, Worthington JR, Hawken S, et al. Using prospective clinical surveillance to identify adverse events in hospital. BMJ Qual Saf 2011;**20**(9):756-63.

20. Lubberding S, Zwaan L, Timmermans DR, et al. The nature and causes of unintended events reported at 10 internal medicine departments. J Patient Saf 2011;**7**(4):224-31.

21. Mason SL, Kuruvilla S, Riga CV, et al. Design and validation of an error capture tool for quality evaluation in the vascular and endovascular surgical theatre. Eur J Vasc Endovasc Surg 2013;**45**(3):248-54.

22. Organisation WH. WHO surgical safety checklist and implementation manual. 2016. http://www.who.int/patientsafety/safesurgery/checklist/en/. Accessed 21st January 2016.

23. Dixon-Woods M, Bosk CL, Aveling EL, et al. Explaining Michigan: developing an ex post theory of a quality improvement program. Milbank Q 2011;**89**(2):167-205.

24. McGinity R, Salokangas M. Introduction: 'embedded research' as an approach into academia for emerging researchers. Management in Education 2014;**28**(1):3-5.

25. Harvey G, Kitson A. PARIHS revisited: from heuristic to integrated framework for the successful implementation of knowledge into practice. Implement Sci 2016;**11**:33.

For peer review only - http://bmjopen.bmj.com/site/about/guidelines.xhtml

26. Checkland K, Snow S, McDermott I, et al. 'Animateurs' and animation: what makes a good commissioning manager? Journal of Health Services Research & Policy 2012;**17**(1):11-17.

27. Moore C, Buchanan DA. Sweat the small stuff: A case study of small-scale change processes and consequences in acute care. Health Services Management Research 2013;**26**(1):9-17.

28. Reynolds M, Jheeta S, Benn J, et al. Improving feedback on junior doctors' prescribing errors: mixed-methods evaluation of a quality improvement project. BMJ Qual Saf 2016;10.1136/bmjqs-2015-004717.

29. Health and Social Care Information Centre. Introduction to Healthcare Resource Groups. http://www.hscic.gov.uk/hrg. Accessed 4th February 2016.

30. Sexton JB, Helmreich RL, Neilands TB, et al. The Safety Attitudes Questionnaire: psychometric properties, benchmarking data, and emerging research. BMC Health Serv Res 2006;**6**:44.

31. Havyer RD, Wingo MT, Comfere NI, et al. Teamwork assessment in internal medicine: a systematic review of validity evidence and outcomes. J Gen Intern Med 2014;**29**(6):894-910.

32. Hemming K, Girling A. A menu-driven facility for power and detectable-difference calculations in stepped-wedge cluster-randomized trials. Stata J 2014;**14**(2):363-80.

33. Kul S, Vanhaecht K, Panella M. Intraclass correlation coefficients for cluster randomized trials in care pathways and usual care: hospital treatment for heart failure. BMC Health Serv Res 2014;**14**:84.

For peer review only

34. Pearse R. Enhanced peri-operative care for high-risk patients (EPOCH) trial: a stepped wedge cluster randomised trial of a quality improvement intervention for patients undergoing emergency laparotomy. http://www.epochtrial.org/docs/EPOCH Protocol revision V2 0 280414.pdf. Accessed 10th December 2014.

35. Hemming K, Taljaard M. Sample size calculations for stepped wedge and cluster randomised trials: a unified approach. J Clin Epidemiol 2016;**69**:137-46.

36. Dimick JB, Ryan AM. Methods for evaluating changes in health care policy: the difference-in-differences approach. JAMA 2014;**312**(22):2401-2.

37. Health and Social Care Information Centre. Indicator Specification: Summary Hospital-level Mortality Indicator methodology (Appendix D, version 1.17). http://www.hscic.gov.uk/media/11151/Indicator-Specification-Summary-Hospital-level-Mortality-Indicator-methodology/pdf/SHMI_Specification.pdf. Accessed 9th October 2014.

38. NASA-Task Load Index (NASA-TLX); 20 Years Later. Proceedings of the Human Factors and Ergonomics Society 50th Annual Meeting; 2006; Santa Monica. HFES.

39. Nicholas LH, Dimick JB. Too much of a good thing? Multi-tasking and distraction in quality improvement. Arch Surg 2011;**146**(3):270.

40. McMullen H, Griffiths C, Leber W, et al. Explaining high and low performers in complex intervention trials: a new model based on diffusion of innovations theory. Trials 2015;**16**:242.

For peer review only

41. Pannick S, Archer S, Johnston MJ, et al. Translating concerns into action: a detailed qualitative evaluation of an interdisciplinary intervention on medical wards. BMJ Open [in press] 2017.

42. Rotter T, Kinsman L, James E, et al. Clinical pathways: effects on professional practice, patient outcomes, length of stay and hospital costs. Cochrane Database Syst Rev 2010;10.1002/14651858.CD006632.pub2(3):CD006632.

43. Dixon-Woods M, Martin G, Tarrant C, et al. Safer Clinical Systems: evaluation findings. London: The Health Foundation, 2014.

44. Pannick S, Davis R, Ashrafian H, et al. Effects of Interdisciplinary Team Care Interventions on General Medical Wards: A Systematic Review. JAMA Intern Med 2015;**175**(8):1288-98.

45. Bhamidipati VS, Elliott DJ, Justice EM, et al. Structure and outcomes of interdisciplinary rounds in hospitalized medicine patients: A systematic review and suggested taxonomy. Journal of Hospital Medicine 2016;10.1002/jhm.2575:n/a-n/a.

46. Mayer EK, Sevdalis N, Rout S, et al. Surgical Checklist Implementation Project: The Impact of Variable WHO Checklist Compliance on Risk-adjusted Clinical Outcomes After National Implementation: A Longitudinal Study. Ann Surg 2016;**263**(1):58-63.

47. O'Leary KJ, Killarney A, Hansen LO, et al. Effect of patient-centred bedside rounds on hospitalised patients' decision control, activation and satisfaction with care. BMJ Qual Saf 2015;10.1136/bmjqs-2015-004561.

48. Loudon K, Treweek S, Sullivan F, et al. The PRECIS-2 tool: designing trials that are fit for purpose. BMJ 2015;**350**:h2147.

For peer review only

49. Needleman J, Buerhaus P, Pankratz VS, et al. Nurse staffing and inpatient hospital mortality. N Engl J Med 2011;**364**(11):1037-45.

50. Griffiths P, Ball J, Murrells T, et al. Registered nurse, healthcare support worker, medical staffing levels and mortality in English hospital trusts: a cross-sectional study. BMJ Open 2016;**6**(2):e008751.

51. Cowen S, Ellison SLR. Reporting measurement uncertainty and coverage intervals near natural limits. Analyst 2006;**131**(6):710-17.

LEGENDS FOR FIGURES

Figure 1: Patient flow diagram

Figure 2: Relationship between engagement and frequency of HEADS-UP briefings, and the probability of excess length of stay

Supplement Figure 1: Ward implementation schedule

Supplement Figure 2: HEADS-UP briefing pro forma

For peer review only

Table 1. Institution characteristics.

	Hospital 1	Hospital 2
Institution type (inpatient admissions / year)		
	Community hospital (52,000)	Academic teaching hospital (186,000)
Participating wards (n)		
	Acute medical unit (2)<br>Gastroenterology/ internal medicine (1)<br>Heart failure/ internal medicine (1)<br>Geriatrics (1)<br>Respiratory / internal medicine (1)	Geriatrics (1)
Existing interdisciplinary practice and approaches to adverse event detection & monitoring		
	Daily interdisciplinary 'board round', typically focused on patients' discharge requirements	Daily interdisciplinary 'board round', typically focused on patients' discharge requirements
	Online incident reporting system	Online incident reporting system
	Unstructured mortality case-note reviews	
Major organisational changes during the study period		
	Incipient institutional merger	Deployment of a new electronic health record

For peer review only

Table 2: Ward and admission characteristics during the intervention period. Values are median (interquartile range) unless otherwise stated.

	Control group	Intervention group
Ward characteristics		
Number of patient admissions	4927	6518
Ward admissions per month	64 (55-153)	77 (64-133)
Length of stay for study patients, days	2.0 (0.8-7.6)	2.2 (0.9-7.3)
Admission characteristics		
Age, years	67 (49-80)	65 (47-78)
Age ≥ 65 years [n, (%)]	2707 (54.9)	3275 (50.2)
Female [n, (%)]	2537 (51.5)	3391 (52.0)
Charlson comorbidity index	3 (0-10)	3 (0-8)
Palliative coding [n, (%)]	50 (1.0)	94 (1.4)

review only

Table 3. Summary of analysis for primary and secondary outcomes.

Outcome	HEADS-UP	Control	Adjusted outcome ratio[§]			
			Intention-to-treat		High fidelity[±]	
			Outcome ratio (95% CI)	*P value*	*Outcome ratio (95% CI)*	*P value*
Primary outcome						
eLOS, no. (%)	1714 (26.3)	1279 (26.0)	**1.32 (1.10-1.58)**	**0.003**	**0.79 (0.67-0.94)**	**0.006**
Secondary outcomes						
Readmission within 30 days, no. (%)	950 (14.6)	565 (11.5)	1.12 (0.91-1.37)	0.296	1.11 (0.93-1.32)	0.247
In-hospital death or death/readmission within 30 days, no. (%)	1657 (24.2)	1190 (23.0)	1.03 (0.86-1.22)	0.759	1.07 (0.92-1.25)	0.363
Escalation events/month, median (IQR)*	8 (3.5-16)	3 (1-6)	0.95 (0.74-1.23)	0.713	1.01 (0.83-1.24)	0.919
Complications of care/month, median (IQR)	1 (0-2)	1 (0-3)	0.92 (0.56-1.50)	0.727	1.28 (0.76-2.13)	0.351
Total incident reports/month, median (IQR)	16 (10-23.5)	15.5 (9.25-20)	0.97 (0.82-1.14)	0.673	**1.28 (1.12-1.47)**	**<0.001**
High yield incident reports/month, median (IQR) **	11 (6-17.5)	9 (5-14.75)	0.92 (0.75-1.12)	0.400	**1.40 (1.19-1.65)**	**<0.001**
Non-nurse incident reports/month, median (IQR)	2 (0.5-4)	1 (1-2.75)	0.89 (0.55-1.42)	0.618	**1.79 (1.24-2.58)**	**0.002**

For peer review only

§ - Odds ratio for binary outcomes; incidence rate ratio for continuous outcomes

± - high fidelity = high frequency (≥75% working days) and monthly reporting ≥ median issues

* - Escalation events = emergency calls, ITU transfers & referrals to the ICU outreach service

** - High yield incident reports = not relating to slips, trips or falls

NB Patient-level outcomes were adjusted for time effects, age, Charlson comorbidity index, and palliation status, as well as ward admissions. Ward-aggregate outcomes were adjusted for time effects, seasonal trends, the median Charlson score of patients on the ward that month, and the rotation of interns between departments.

For peer review only

Table 4: Effect of PCTS on safety and teamwork attitudes

		PCTS		*P value*
		**Participants**	**Non-participants**	
Mean safety score (SD)		68.4 (13.6)	65.9 (16.9)	0.667
Mean teamwork score (SD)		80.2 (12.1)	79.6 (12.3)	0.524
Estimated marginal mean safety score [95% CI]				**0.021***
Hospital 1	Baseline	59.8 [51.4-68.1]	69.2 [64.6-73.9]	
	6 months	70.3 [65.3-75.2]	61.0 [50.8-71.2]	
Hospital 2	Baseline	70.8 [51.1-90.4]	54.9 [46.2-63.7]	
	6 months	71.0 [56.0-86.0]	75.1 [63.5-86.7]	
Estimated marginal mean teamwork score [95% CI]				**0.004***
Hospital 1	Baseline	72.3 [65.3-79.2]	82.6 [78.8-86.5]	
	6 months	81.7 [77.6-85.8]	70.0 [61.6-78.4]	
Hospital 2	Baseline	84.8 [68.5-100.0**]	74.5 [67.2-81.7]	
	6 months	88.2 [75.7-100.0**]	83.3 [73.7-92.9]	

* *p* value for model effect of interaction term PCTS participation*hospital*time

** The upper bound of the 95% CI for estimated marginal mean climate scores was truncated at 100, the limit of the scale.[51]

For peer review only

For peer review only

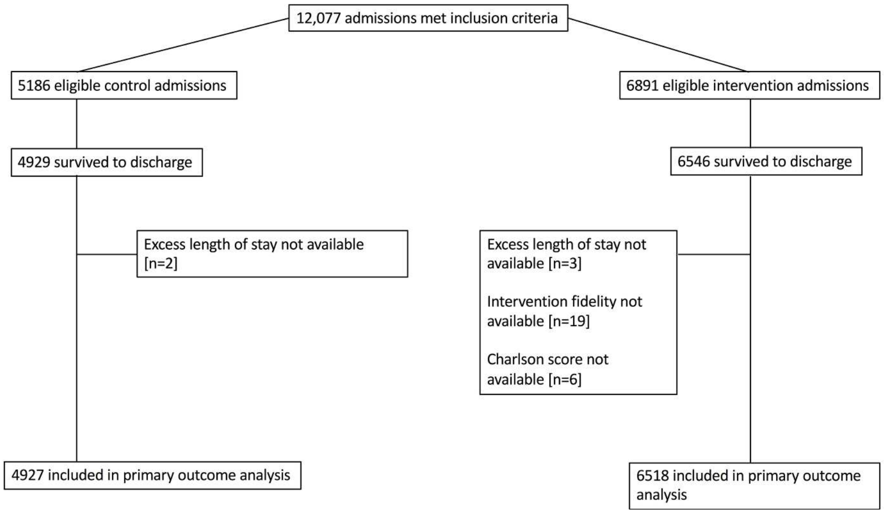

Figure 1: Patient flow diagram

209x148mm (300 x 300 DPI)

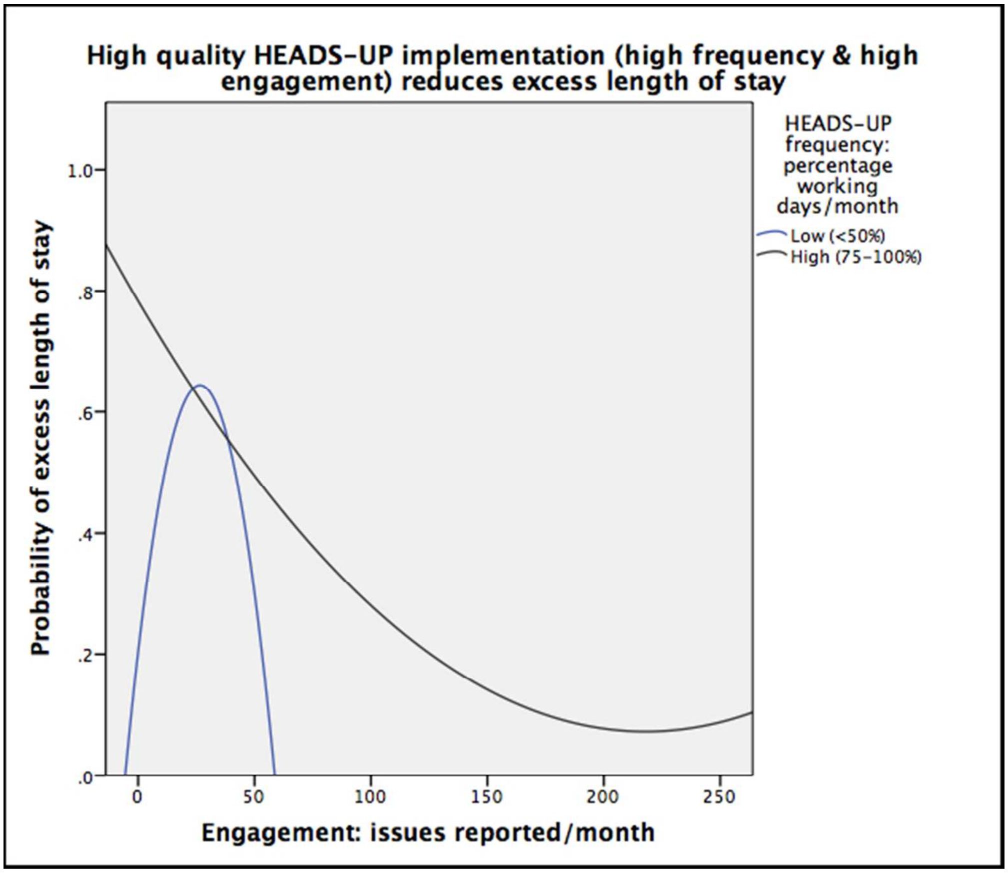

Figure 2: Relationship between engagement and frequency of HEADS-UP briefings, and the probability of excess length of stay

221x191mm (72 x 72 DPI)

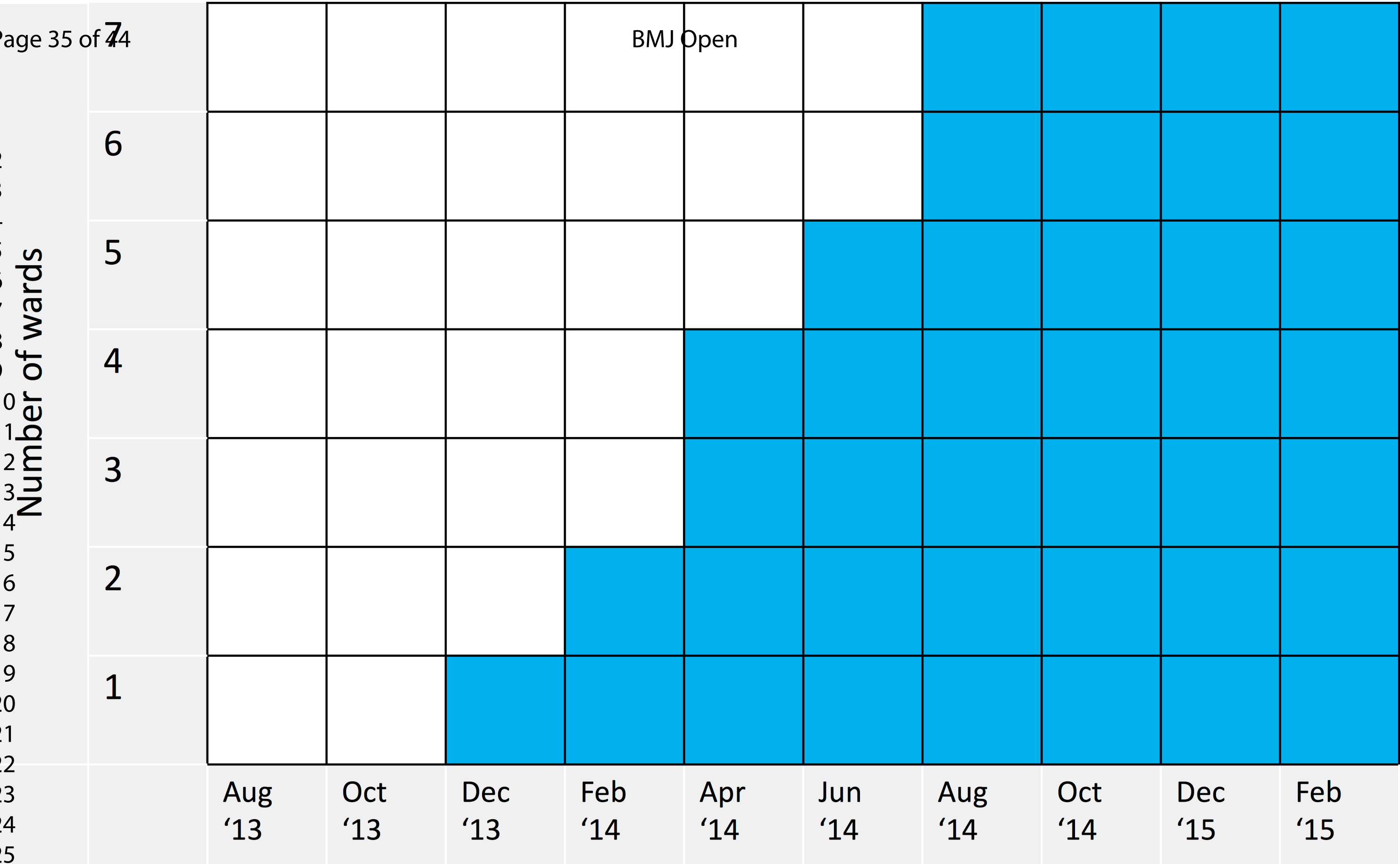
Number of wards
7
6
5
4
3
2
1
Aug ‘13
Oct ‘13
Dec ‘13
Feb ‘14
Apr ‘14
Jun ‘14
Aug ‘14
Oct ‘14
Dec ‘15
Feb ‘15

What happened yesterday?

HEADS-UP

A first class hospital for our community

Design by HELIX
helixcentre.com

If a patient has been affected, please include their hospital number. Where there is nothing to report, write N/A in comments box.

1 Review details

Ward:

Scribe name: Role:

Date:

Team members present

- Clerk
- HCA
- Physio
- OT
- Nurse
- Pharmacist
- Discharge team
- Consultant
- SpR
- CT
- F2
- F1

2 Concerns

Staffing levels

- Medical
- Nursing
- OT / PT

Comment or N/A

Equipment

- Unavailable
- Not working

Comment or N/A

Services

- Phlebotomy
- Echo
- Radiology
- Endoscopy
- In-patient review
  - Delayed
  - Too junior

Comment or N/A

Missed results / diagnosis

Comment or N/A

3 Events

Handover / communication problem

Comment or N/A

New hospital-acquired problem

- Pressure ulcer
- Pneumonia
- UTI
- MRSA
- C-Diff
- Other

Comment or N/A

Patient deterioration

- Sepsis
- Delirium
- Fall
- Arrhythmia
- Seen by medical team
- Seen by outreach / ICU

Comment or N/A

Death

- Expected
- Unexpected

Comment or N/A

Patient not discharged as expected

Comment or N/A

4 Drug errors and other issues

- Prescription
- Administration
- VTE
- Analgesia
- Antibotics
- Nutrition

Comment or N/A

5 What went really well?

Comment or N/A

6 Who will...

Arrange senior review / outreach for sick patient?	Name or N/A
Change a prescription?	Name or N/A
Escalate equipment problem / discharge delay?	Name or N/A
Complete a DATIX?	Name or N/A
Log patient for M&M discussion?	Name or N/A
Other?	Name or N/A

For peer review only - http://bmjopen.bmj.com/site/about/guidelines.xhtml

Supplement Table 1: survey respondents and completion rates

Variable	Baseline	6 months
Professional background [n (%)]		
Consultant physician	8 (13.1)	5 (9.3)
Junior physician	28 (45.9)	35 (64.8)
Senior nurse	15 (24.6)	7 (13.0)
No answer	10 (16.4)	7 (13.0)
Median time on the ward [months]		
	4-6	0-3
Ward specialty [n (%)]		
Acute medicine	19 (31.1)	10 (18.5)
Gastroenterology & general medicine	13 (21.3)	11 (20.4)
Heart failure & general medicine	4 (6.6)	5 (9.3)
Geriatrics	16 (26.2)	14 (25.9)
Respiratory & general medicine	9 (14.8)	12 (22.2)
No answer	0	2 (3.7)
Participated in more than 5 HEADS-UP briefings [n (%)]		
Yes	13 (21.3)	37 (68.5)
No	47 (77.0)	14 (25.9)
No answer	1 (1.6)	3 (5.6)
Workload perception score (NASA-TLX) [mean (SD)]		
	13.2 (2.7)	12.8 (3.1)
Unscored safety and teamwork items [n (%)]		
	33/1647 (2.0)	37/1458 (2.5)
Unscored NASA-TLX items [n (%)]		
	21/366 (5.7)	(18/324) (5.6)

For peer review only

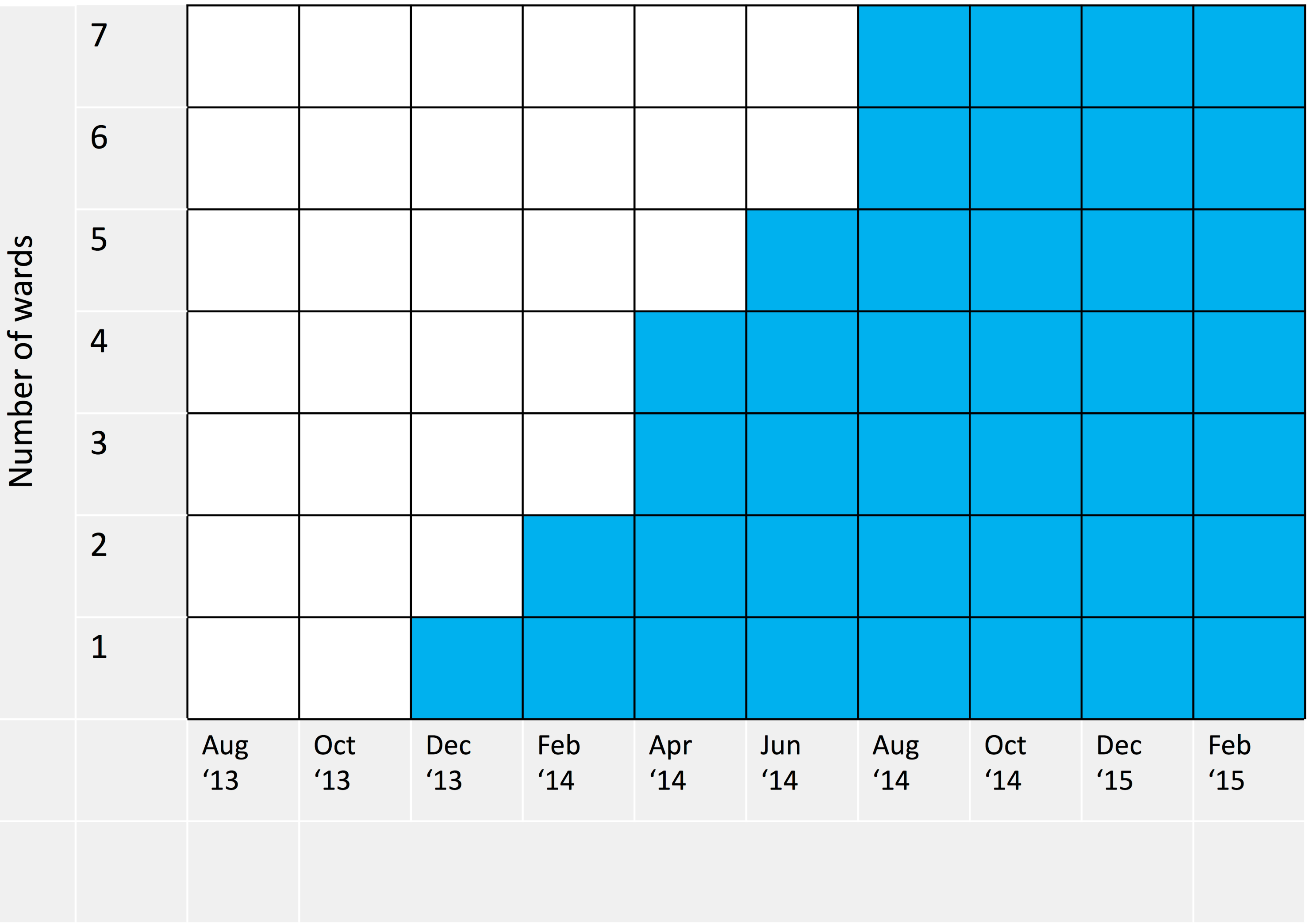
Number of wards
7
6
5
4
3
2
1
Aug ‘13
Oct ‘13
Dec ‘13
Feb ‘14
Apr ‘14
Jun ‘14
Aug ‘14
Oct ‘14
Dec ‘15
Feb ‘15

Section/Topic	Item No	Standard Checklist item	Extension for cluster designs	Page No *
**Title and abstract**				
	1a	Identification as a randomised trial in the title	Identification as a cluster randomised trial in the title	1
	1b	Structured summary of trial design, methods, results, and conclusions (for specific guidance see CONSORT for abstracts)[1,2]	See table 2	3
**Introduction**				
**Background and objectives**	2a	Scientific background and explanation of rationale	Rationale for using a cluster design	7
	2b	Specific objectives or hypotheses	Whether objectives pertain to the the cluster level, the individual participant level or both	8
**Methods**				
**Trial design**	3a	Description of trial design (such as parallel, factorial) including allocation ratio	Definition of cluster and description of how the design features apply to the clusters	7
	3b	Important changes to methods after trial commencement (such as eligibility criteria), with reasons		N/A
**Participants**	4a	Eligibility criteria for participants	Eligibility criteria for clusters	7
	4b	Settings and locations where the data were collected		7
**Interventions**	5	The interventions for each group with sufficient details to allow replication, including how and when they were actually administered	Whether interventions pertain to the cluster level, the individual participant level or both	5-7
**Outcomes**	6a	Completely defined pre-specified primary and secondary outcome measures, including how and	Whether outcome measures pertain to the cluster level, the individual participant level or both	8

		when they were assessed		
	6b	Any changes to trial outcomes after the trial commenced, with reasons		N/A
**Sample size**	7a	How sample size was determined	Method of calculation, number of clusters(s) (and whether equal or unequal cluster sizes are assumed), cluster size, a coefficient of intracluster correlation (ICC or *k*), and an indication of its uncertainty	8
	7b	When applicable, explanation of any interim analyses and stopping guidelines		N/A
**Randomisation:**				
**Sequence generation**	8a	Method used to generate the random allocation sequence		7
	8b	Type of randomisation; details of any restriction (such as blocking and block size)	Details of stratification or matching if used	7
**Allocation concealment mechanism**	9	Mechanism used to implement the random allocation sequence (such as sequentially numbered containers), describing any steps taken to conceal the sequence until interventions were assigned	Specification that allocation was based on clusters rather than individuals and whether allocation concealment (if any) was at the cluster level, the individual participant level or both	8
**Implementation**	10	Who generated the random allocation sequence, who enrolled participants, and who assigned participants to interventions	Replace by 10a, 10b and 10c	
	10a		Who generated the random allocation sequence, who enrolled clusters, and who assigned clusters to interventions	7
	10b		Mechanism by which individual participants were included in clusters for the purposes of the	7

			trial (such as complete enumeration, random sampling)	
	10c		From whom consent was sought (representatives of the cluster, or individual cluster members, or both), and whether consent was sought before or after randomisation	7
**Blinding**	11a	If done, who was blinded after assignment to interventions (for example, participants, care providers, those assessing outcomes) and how		8
	11b	If relevant, description of the similarity of interventions		N/A
**Statistical methods**	12a	Statistical methods used to compare groups for primary and secondary outcomes	How clustering was taken into account	9
	12b	Methods for additional analyses, such as subgroup analyses and adjusted analyses		9
**Results**				
**Participant flow (a diagram is strongly recommended)**	13a	For each group, the numbers of participants who were randomly assigned, received intended treatment, and were analysed for the primary outcome	For each group, the numbers of clusters that were randomly assigned, received intended treatment, and were analysed for the primary outcome	10
	13b	For each group, losses and exclusions after randomisation, together with reasons	For each group, losses and exclusions for both clusters and individual cluster members	Supplement
**Recruitment**	14a	Dates defining the periods of recruitment and follow-up		7
	14b	Why the trial ended or was stopped		7
**Baseline data**	15	A table showing baseline	Baseline characteristics for the	19

		demographic and clinical characteristics for each group	individual and cluster levels as applicable for each group	
**Numbers analysed**	16	For each group, number of participants (denominator) included in each analysis and whether the analysis was by original assigned groups	For each group, number of clusters included in each analysis	Supplement
**Outcomes and estimation**	17a	For each primary and secondary outcome, results for each group, and the estimated effect size and its precision (such as 95% confidence interval)	Results at the individual or cluster level as applicable and a coefficient of intracluster correlation (ICC or k) for each primary outcome	10-11
	17b	For binary outcomes, presentation of both absolute and relative effect sizes is recommended		10-11
**Ancillary analyses**	18	Results of any other analyses performed, including subgroup analyses and adjusted analyses, distinguishing pre-specified from exploratory		10-11
**Harms**	19	All important harms or unintended effects in each group (for specific guidance see CONSORT for harms[3])		N/A
**Discussion**				
**Limitations**	20	Trial limitations, addressing sources of potential bias, imprecision, and, if relevant, multiplicity of analyses		11-13
**Generalisability**	21	Generalisability (external validity, applicability) of the trial findings	Generalisability to clusters and/or individual participants (as relevant)	13
**Interpretation**	22	Interpretation consistent with results, balancing benefits and harms, and considering other relevant evidence		11-13
**Other information**				

For peer review only

**Registration**	23	Registration number and name of trial registry	4
**Protocol**	24	Where the full trial protocol can be accessed, if available	7
**Funding**	25	Sources of funding and other support (such as supply of drugs), role of funders	2

** Note: page numbers optional depending on journal requirements*

For peer review only

For peer review only
